# Characterization of immune responses to anti-PD-1 mono and combination immunotherapy in hematopoietic humanized mice implanted with tumor xenografts

**DOI:** 10.1186/s40425-019-0518-z

**Published:** 2019-02-08

**Authors:** A. Capasso, J. Lang, T. M. Pitts, K. R. Jordan, C. H. Lieu, S. L. Davis, J. R. Diamond, S. Kopetz, J. Barbee, J. Peterson, B. M. Freed, B. W. Yacob, S. M. Bagby, W. A. Messersmith, J. E. Slansky, R. Pelanda, S. G. Eckhardt

**Affiliations:** 1 Division of Medical Oncology, School of Medicine, University of Colorado, Anschutz Medical Campus, 13001 E 17th Pl, Aurora, CO 80045 USA; 2 University of Colorado Cancer Center, University of Colorado, Anschutz Medical Campus, 1665 Aurora Ct, Aurora, CO 80045 USA; 3 Department of Immunology and Microbiology, School of Medicine, University of Colorado, Anschutz Medical Campus, 12800 E. 19th Ave P18-8401G, 13001 E 17th Pl, Aurora, CO 80045 USA; 4 MD Anderson Cancer Center, 1515 Holcombe Blvd10, Houston, TX 77030 USA; 5 Department of Oncology, Dell Medical School, The University of Texas at Austin, 1701 Trinity Street, Austin, TX 78712 USA; 6 Division of Allergy and Clinical Immunology, School of Medicine, University of Colorado Denver, 13001 E 17th Pl, Aurora, CO 80045 USA

**Keywords:** Humanized mice, Immunotherapy, Nivolumab, Combination, Pre-clinical, PDX, CRC, TNBC

## Abstract

**Background:**

The success of agents that reverse T-cell inhibitory signals, such as anti-PD-1/PD-L1 therapies, has reinvigorated cancer immunotherapy research. However, since only a minority of patients respond to single-agent therapies, methods to test the potential anti-tumor activity of rational combination therapies are still needed. Conventional murine xenograft models have been hampered by their immune-compromised status; thus, we developed a hematopoietic humanized mouse model, hu-CB-BRGS, and used it to study anti-tumor human immune responses to triple-negative breast cancer (TNBC) cell line and patient-derived colorectal cancer (CRC) xenografts (PDX).

**Methods:**

BALB/c-Rag2^null^Il2rγ^null^SIRPα^NOD^ (BRGS) pups were humanized through transplantation of cord blood (CB)-derived CD34+ cells. Mice were evaluated for human chimerism in the blood and assigned into experimental untreated or nivolumab groups based on chimerism. TNBC cell lines or tumor tissue from established CRC PDX models were implanted into both flanks of humanized mice and treatments ensued once tumors reached a volume of ~150mm^3^. Tumors were measured twice weekly. At end of study, immune organs and tumors were collected for immunological assessment.

**Results:**

Humanized PDX models were successfully established with a high frequency of tumor engraftment. Humanized mice treated with anti-PD-1 exhibited increased anti-tumor human T-cell responses coupled with decreased Treg and myeloid populations that correlated with tumor growth inhibition. Combination therapies with anti-PD-1 treatment in TNBC-bearing mice reduced tumor growth in multi-drug cohorts. Finally, as observed in human colorectal patients, anti-PD-1 therapy had a strong response to a microsatellite-high CRC PDX that correlated with a higher number of human CD8+ IFNγ+ T cells in the tumor.

**Conclusion:**

Hu-CB-BRGS mice represent an in vivo model to study immune checkpoint blockade to human tumors. The human immune system in the mice is inherently suppressed, similar to a tumor microenvironment, and thus allows growth of human tumors. However, the suppression can be released by anti-PD-1 therapies and inhibit tumor growth of some tumors. The model offers ample access to lymph and tumor cells for in-depth immunological analysis. The tumor growth inhibition correlates with increased CD8 IFNγ+ tumor infiltrating T cells. These hu-CB-BRGS mice provide a relevant preclinical animal model to facilitate prioritization of hypothesis-driven combination immunotherapies.

**Electronic supplementary material:**

The online version of this article (10.1186/s40425-019-0518-z) contains supplementary material, which is available to authorized users.

## Background

Development of agents that target immune regulatory checkpoints, such as CTLA-4 and PD-1/PD-L1, have revolutionized cancer treatments [1]. Blockade of immune checkpoints has led to substantial clinical success with durable objective tumor regression and prolonged survival in several malignancies [2–8]. However, not all patients respond to these therapies [9]. Combining checkpoint therapies with these and other immunotherapies, small molecules, epigenetic modifiers, or targeted cancer drugs may improve outcomes [10] but immune-competent model systems to identify and prioritize appropriate combinations are limited.

Recently ex vivo organotypic microfluidic, spheroid culture models have been utilized to screen and identify small molecules that can be used in combination strategies to enhance efficacy of exisiting immunotherapies [11–14]. However, these approaches are hampered by a lack of dynamic interactions between the tumor, tumor microenvironment (TME), and immune system and an inability to investigate in vivo conditions that may influence the tumor-immune system interaction [11, 15]. Similarly, the use of syngeneic murine-derived cancer models in immune-competent mice presents significant limitations because of the inconsistencies between murine and human immune systems (HIS) and the limited repertoire of available syngeneic models [16–18]. Thus, better in vivo preclinical models, which harbor a HIS and are capable of growing more diverse patient-derived human tumors, are needed to test immunotherapeutic approaches. Hematopoietic humanized mice are generated by intravenous injection of human CD34+ stem cells derived from umbilical cord blood (CB) into immunodeficient mice lacking T, B and NK cells [19–22]. These mice have been shown to accept and grow tumor cells from patient derived xenografts (PDXs) [23].

In the current study, we used an hematopoietic humanized mouse model, hu-CB-BRGS, which has been extensively optimized and characterized in our lab, as a model to study tumor immunotherapies [19, 20, 24]. We show that the T-cells of hu-CB-BRGS express high levels of PD-1, suggesting an immunosuppressed, exhausted phenotype. This is validated by vigorous engraftment of non-HLA matched human tumors. Using hu-CB-BRGS mice, we demonstrated that a triple negative breast cancer (TNBC) cell line is more effectively treated with anti-PD-1 immunotherapy and an epigenetic modifier than either treatment alone. Using patient-derived colorectal cancer xenografts (CRC PDX), we also demonstrated that the immune system responds differentially to microsatellite instable-high (MSI-H) and microsatellite stable (MSS) tumors, consistent with data reported from clinical trials [9]. We propose that this humanized PDX mouse model can be used to test rational combination therapies designed to enhance the efficacy of PD-1 blockade and allow an in-depth study of both the immune response and tumor microenvironment in a mouse host with a HIS.

## Methods

### Generation of humanized mice

Isolation of human CD34+ cells from CB and generation of hu-CB-BRGS mice (Additional file 1) were performed as described previously [19, 20]. Investigators were blinded from donor identities, and the studies were performed in compliance with University of Colorado Institutional Review Board.

### Tumor injections, evaluations and drug treatments

The MDA-MB-231 cell line purchased from American Type Culture Collection was used to model TNBC. Cells were authenticated by the Barbara Davis Center for Childhood Diabetes Core and tested for absence of mycoplasma every 3 months. Cells used for in vivo studies were passaged 3 times prior to injection into mice. Cells were harvested during exponential growth and resuspended in a 1:1 mixture of DMEM supplemented with 10% FBS, 1% PenStrep and 1% non essential amino acids and Matrigel (BD Biosciences). Five to ten million cells per mouse were injected subcutaneously into both flanks using a 23-gauge needle [25].

For the colorectal models, the PDX tumors were generated and grown in nude mice prior to implantation in hu-CB-BRGS mice, as previously described ([25, 26] and Additional file 1).

Upon verification of human T-cell chimerism in the hu-CB-BRGS mice, tumors were implanted into both hind flanks of 16–21-week old humanized mice or into non-humanized BRGS controls for MDA-MB-231 and MSI-H. When the average tumor size reached a volume of approximately 150–300 mm^3^, experimental treatments were begun. Nivolumab, anti-PD-1, was administered intraperitoneally (i.p.) at approximately 30 mg/kg twice per week. For combination therapy studies, OKI-179, a histone deacetylase (HDAC) inhibitor, was administered via oral gavage at 30 mg/kg three times per week and nivolumab was administrated i.p. 30 mg/kg d0–10, then 15 mg/kg after d10 once a week. Control mice were left untreated. Twice weekly, mice were monitored for signs of toxicity and tumors were measured using calipers, shaving mice when necessary, to better visualize tumors (Additional file 1).

### Chimerism evaluation, tissue harvest, cell staining and flow cytometry

Evaluation of human chimerism in the blood is described in Additional file 1.

Euthanasia was performed either at end of study or when total tumor burden approached 3000 mm^3^ (typically 20–45 d). The detailed timepoints for each mouse in each experiment are provided in Additional file 2. Blood was collected from the heart immediately following euthanasia and sera were frozen for future analyses. Lymph nodes (LNs), spleen, and bone marrow (BM) tissues were processed into single-cell suspensions as described previously [19, 20]. Tumors were extracted, weighed and a small portion was fixed in formalin for histological analyses. The remaining tumor sections were minced and digested for 25 min at 37 °C in Liberase DL (50 μg/ml) containing RPMI serum-free medium. Following incubation, the tissue was strained through a 100 μm filter, and the filter was washed with 5–10 ml of complete RPMI medium containing 10% heat-inactivated FBS, 100 U/ml penicillin, 100 μg/ml streptomycin and non-essential amino acids to stop digestion. The tumor cells were resuspended in fresh DNAase-containing Iscove’s medium with 5% heat-inactivated FCS, Hepes and Glutamax. Cells from the spleen and LNs were counted using a hemocytometer and samples from all tissue preparations were collected for a set time on the flow cytometer to generate a relative cell count.

As described previously, single cell suspensions were incubated with fluorescently-labeled antibodies (Abs, see Additional file 3) to evaluate immune parameters described in Additional file 1.

### Multispectral imaging of tumors

Upon dissection, a portion of the tumor was fixed in formalin and paraffin-embedded for multispectral imaging on Vectra 3.0 Automated Quantitative Pathology Imaging System (Perkin Elmer, described in Additoinal file 1).

### Elisa

Concentrations of human IgM and IgG in the sera of humanized mice were measured as described previously using a monoclonal mouse anti-human IgM or IgG-based ELISA to avoid detection of the anti-PD-1 drug in the sera [27].

### Statistical analysis

The tumor growth inhibition (TGI) was calculated from the average tumor volume in treated (Vt) and vehicle control (Vvc) groups, according to the following equation: TGI = 100 × (Vt_final_ - Vt_initial_)/(Vvc_final_ -Vvc_intial_). Statistical significance of immune measurements were assessed using Prism software (GraphPad software) with a two-tailed Student’s t-test of equal variance or Welch’s correction when appropriate. *P*-values less than 0.05 were considered significant.

## Results

### Nivolumab treatment inhibits growth of the TNBC MDA-MB-231 cell line in hu-CB-BRGS mice

**Fig. 1:**
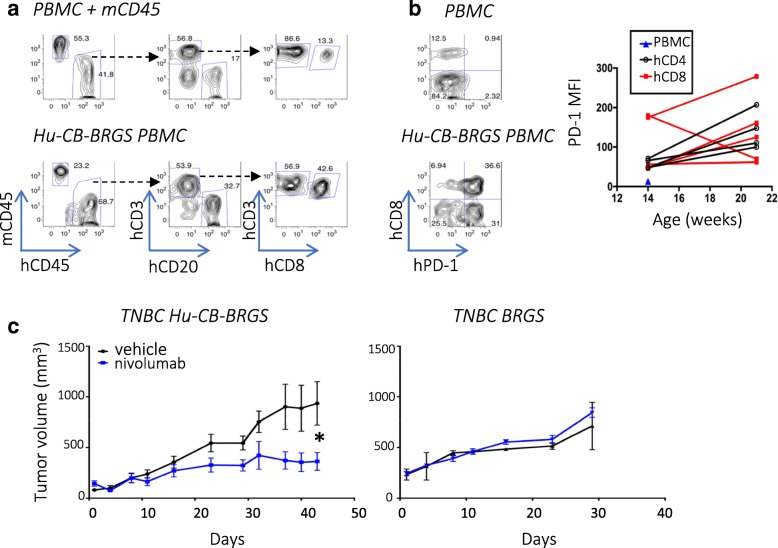
Anti-PD-1 therapy inhibits growth of TNBC xenograft in hu-CB-BRGS model. **a**, PBMCs of humanized mice were analyzed pre-tumor engraftment for expression of mouse (mCD45) and human (hCD45) hematopoietic markers (left panels). The hCD45+ cells were analyzed for expression of T-cell (CD3), B-cell (CD20), and CD8+ T-cell subsets (middle, right panels). A mixture of CB PBMCs and mouse spleen cells served as a positive staining control (top panels). **b**, Expression of PD-1 on T-cells (hCD45+ CD3+) in PBMCs from a control CB (top) and a 16-week old humanized mouse (bottom). The MFI of PD-1 on CD4+ (black) and most CD8+ (red) T-cells increase with age (right, CD4: *P* = 0.04; CD8: ns). Chimerism and PD-1 staining results represent > 100 hu-CB-BRGS mice. **c**, Growth curves of TNBC MDA-MB-231 tumors in nivolumab-treated (blue line) or untreated (black line) hu-CB-BRGS mice (left, *n* = 6 tumors/3 mice per group) and non-humanized BRGS mice (right, *n* = 2 tumors per group). *P*-values: * < 0.05

We have previously reported that BRG(S) mice are efficiently reconstituted with human CD34+ cells from CB shortly after birth [19] and that develop, with time, a significant population of human B and T cells [20, 24] (Fig. 1a). In this study, we found T-cells from hu-CB-BRGS mice expressed high levels of PD-1, levels that increased with age (Fig. 1b).

With the ultimate goal of using hu-CB-BRGS mice to test anti-tumor immunotherapies and combination therapies, we developed a protocol to engraft tumors and test treatments in these animals (Additional file 4: Figure S1). As proof of concept, we first engrafted hu-CB-BRGS mice with a TNBC cell line and tested the ability of nivolumab, an anti-PD-1 Ab, to stimulate the immune system to inhibit its growth. Specifically, we evaluated MDA-MB-231, a cell line with high PD-L1 expression [28] (Additional file 4: Figure S2), which has recently been reported to respond to immunotherapy in a NSG humanized mouse model [23, 28]. In the hu-CB-BRGS model, greater than 95% of mice injected with MDA-MB-231 cell lines grew detectable tumors (data not shown). Anti-PD-1 monotherapy inhibited tumor growth compared to untreated controls (TGI = 61%, Fig. 1c, left). No response was observed in immunodeficient non-humanized BRGS mice, suggesting the HIS is capable of an anti-tumor response in this model (TGI = − 19%, Fig. 1c right**).**

### Characterization of human T cells in Hu-CB-BRGS mice bearing TNBC tumors in the presence or absence of anti-PD-1 therapy

**Fig. 2:**
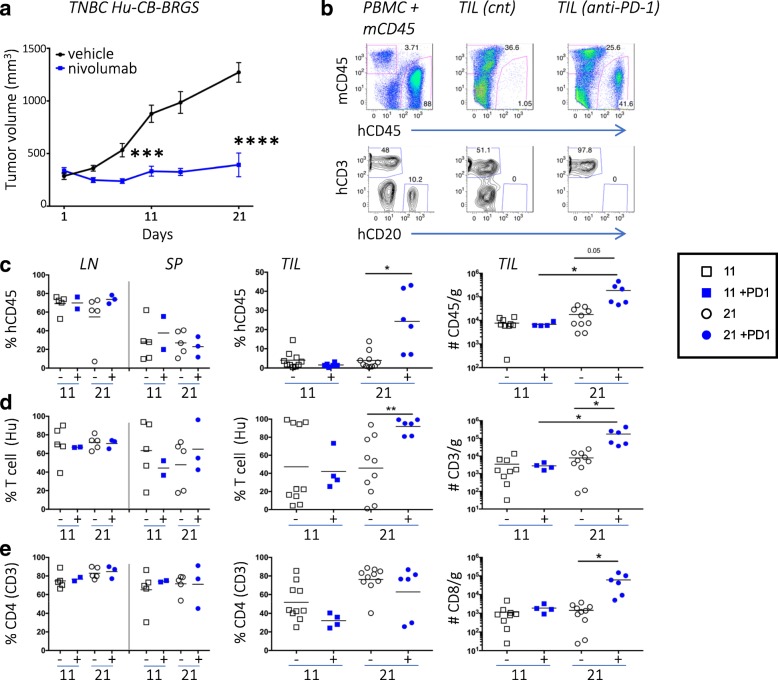
Human T-cell chimerism in lymph organs and tumors of TNBC mice model **a**, Growth of MDA-MB-231 tumors in hu-CB-BRGS mice treated (blue line) or untreated (black line) with nivolumab is shown. **b**, Representative flow plots illustrate gating strategy used to determine human T- and B-cell chimerism. Mouse (mCD45) and human (hCD45) Abs were used to detect species-specific hematopoietic cells (top panels) and hCD3 and hCD20 Abs identified T and B cells, respectively, among the hCD45+ gated cells (bottom panels). A mixture of CB (hPBMC) and mouse spleen cells served as positive staining controls and representative flow plots are from TILs of control or anti-PD-1 treated hu-CB-BRGS mice. 10 tumors were analyzed on d11 and d21 in the control group; 4 tumors on d11 and 6 tumors on d21 were analyzed in the anti-PD-1-treated group. Human immune cell (**c**), T-cell (**d**) and CD4 T-cell (**e**) chimerism in lymph organs and tumors of hu-CB-BRGS mice. CD4+ T-cells are gated on hCD45 + hCD3+. CD8 T-cells are calculated as CD4-negative T-cells. Left panels show percentages in lymph organs, middle panels show frequencies and right panels show relative cell numbers in TILs. Each point represents data from the indicated organ from an individual hu-CB-BRGS mouse that was either untreated (−, black) or treated with nivolumab (+, blue) for 11 or 21 days. *P*-values: * < 0.05, **** < 0.0001

To characterize immune responses to the TNBC cell line in the hu-CB-BRGS model, we engrafted hu-CB-BRGS mice with MDA-MB-231 to examine hCD45+ cells 11 and 21 days post-treatment. Prior to tumor implantation, mice with similar levels of human hematopoietic and T-cell chimerism in the blood were stratified into treatment groups to minimize engraftment effects (Additional file 4: Figure S3a). Again, we observed tumor growth inhibition in the anti-PD-1-treated group compared to untreated controls (Fig. 2a). We next examined both peripheral immune tissues (LN, spleen, BM) as well as tumor-infiltrating leukocytes (TILs) in untreated and anti-PD-1-treated hu-CB-BRGS mice by flow cytometry to evaluate human T, B and myeloid subsets, which have been shown to differ from the human chimerism in the blood and to change over time [20].

Using this flow cytometry approach, we observed an increased frequency and number of human cells, and more significantly human T-cells, in the tumors of the anti-PD-1-treated mice 21 days after the start of treatment (Figs. 2b-d, Additional file 4: Figure S3b). These differences were not observed in the LNs, spleen or BM, nor in any tissue 11 days after treatment (Figs. 2c-d, Additional file 4: Figure S3b). There was no significant bias in the frequencies of CD4 or CD8 subsets among the T-cells, except for an early infiltration of CD8 T-cells in the tumors of both control and anti-PD-1-treated mice (Fig. 2e). In all other lymph organs the CD4 T-cells represented the majority of T-cells, as they often do in mouse and human [29], and were typically > 70% of the T-cell compartment in humanized mice (Fig. 2e). Importantly, the number of CD8 T-cells significantly increased in the TILs of treated mice at day 21 (Fig. 2e).

To examine the location of immune cells within the tumors, we also quantified hematopoietic infiltrates using multispectral imaging. In agreement with the flow cytometry data, we found increased T-cells in treated mice at d21, with an increased density in the intra-tumor stroma (Additional file 4: Figure S4).

**Fig. 3:**
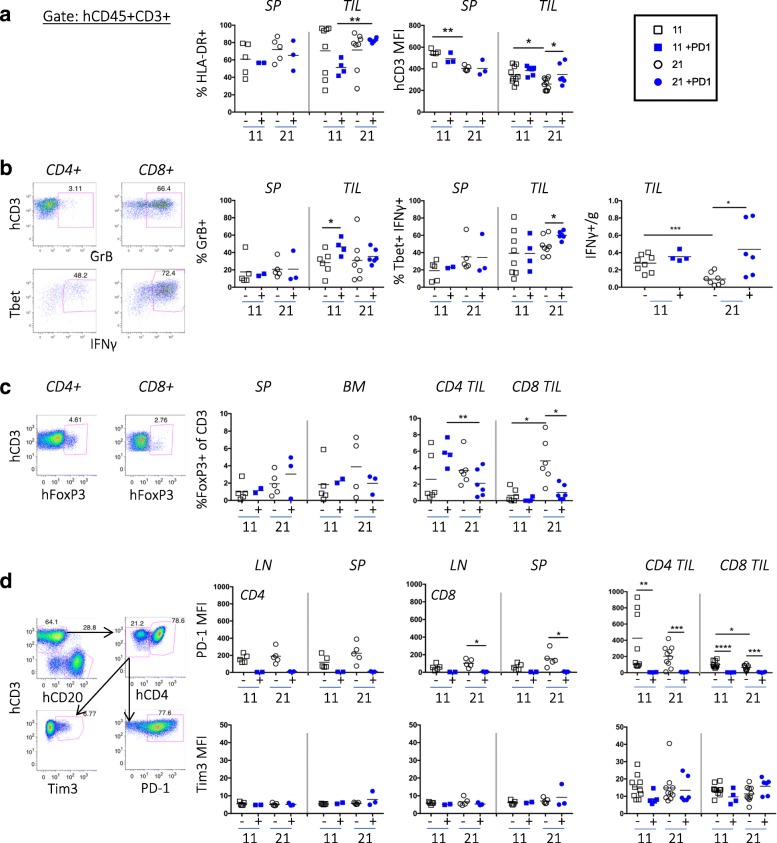
T-cell activation and cytotoxic function in TILs from TNBC-bearing hu-CB-BRGS mice treated with nivolumab***.*** **a**, Quantification of human T-cells (hCD45 + CD3+) that were stained for expression of activation-marker HLA-DR (left) or CD3 (right). **b**, Representative flow cytometric analyses showing gating strategy for identification of GrB and IFNγ secreting T-cells. Frequency of GrB+ (left), Tbet+IFNg+ (middle) and number of IFNγ+ (right) among human T-cells. **c**, Representative flow cytometric analyses (left) showing gating strategy for identification of Treg (FoxP3+) cells and data from each tissue is shown in graphs (right). **d**, Representative flow cytometric analyses showing gating strategy to measure expression levels of human PD-1 and Tim3 inhibitory receptors on T-cells from hu-CB-BRGS mice. The MFI expression of PD-1 and Tim3 is determined for both CD4 and CD8 T-cell subsets and graphed in top (PD-1) and bottom (Tim3) panels. Each dot represents data from the indicated organ from an individual hu-CB-BRGS mouse that was either untreated (−, black) or treated with nivolumab (+, blue) for 11 or 21 days, as indicated. *P*-values: * < 0.05, ** < 0.01, *** < 0.001, **** < 0.0001

To evaluate the activation state of T cells, we compared the expression of T-cell activation markers (CD69, HLA-DR, and CD3) in both the peripheral organs and tumors of hu-CB-BRGS mice. We observed no differences in the percentage of CD69+ T-cells among treated and untreated mice, although a high percentage of both CD4+ and CD8+ T-cells expressed CD69 in all peripheral lymph organs (data not shown, LN: 47–67%, SP:13–74%) and tumors (70–97%). Similarly, a majority of T-cells expressed HLA-DR and the frequency of HLA-DR+ T cells significantly increased in anti-PD-1-treated mice from day 11 to 21 in the TIL (Fig. 3a). Finally, CD3 expression increased on both CD4+ and CD8+ TILs in the treated group relative to the control group on day 21 (Fig. 3a).

To assess T-cell functionality, we stained single cell suspensions of both lymphoid tissues and TILs for intracellular Granzyme B (GrB) and IFNγ, effector molecules associated with T-cell killing (Fig. 3b). Although we detected few GrB+ T-cells in the spleens of any mouse, we found more GrB+ T-cells in the tumors, especially in the nivolumab-treated tumors at day 11 (Fig. 3b). Notably, the percentage and number of IFNγ secreting TILs of anti-PD-1-treated mice significantly increased at day 21 (Fig. 3b). Although both CD4 and CD8 T-cells produced IFNγ, more CD8 T-cells were IFNγ+ than CD4 T-cells (Fig. 3b, data not shown). In addition, the IFNγ+ T-cells expressed high levels of Tbet, a transcription factor associated with Th1 T-cell differentiation, and CD44, a memory and activation marker (Fig. 3b, data not shown).

Using the transcription factor FoxP3 to quantitate human Tregs in humanized mice, we observed 0.2–8% Tregs in the BM and spleen, and their frequency did not change over time or with treatment (Fig. 3c). We confirmed co-expression of CD25 on both CD4+ and CD8+ FoxP3+ T cells in the majority (> 60%) of the Tregs in our hu-CB-BRGS mice (Additional file 4: Figure S5). Notably, we observed more Tregs (CD4 and CD8) among the TILs of both treated and untreated mice, and this frequency increased over time in control mice but not in the TILs of the anti-PD-1 treated mice (Fig. 3c). Altogether, in the tumors of anti-PD-1 treated mice, we detect increased frequencies and numbers of CD8+ TILs with high frequencies of activation markers and IFNγ production, upregulation of CD3 and decreased Treg (FoxP3+) populations, consistent with a more potent, destructive immune response.

To determine if nivolumab treatment changed the expression of inhibitory receptors on human T-cells, we used flow cytometry to measure the expression of PD-1 and Tim3 on human CD4 and CD8 T-cells (Fig. 3d). The PD-1 receptor was much higher in the TILs than peripheral lymph organs in untreated hu-CB-BRGS mice. However PD-1 was barely detectable on T-cells from all tissues in nivolumab-treated mice (Fig. 3d), suggesting the drug saturated the PD-1 receptors. This lack of detectable PD-1 following drug has been reported in other humanized mouse studies [23] as well as melanoma patient studies in which they were able to detect bound Pembolizumab with an anti-human IgG4 Ab [30]. We have not been able to detect PD-1 following treatment with either nivolumab or pembrolizumab, or using two distinct monoclonal anti-PD-1 or polyclonal detecting antibodies, despite reports of distinct binding sites for these two anti-PD-1 drugs [31]. Notably, Tim3 expression was higher on tumor-infiltrating T cells than T-cells from LNs and spleen (Fig. 3d). However, there were no significant differences in the expression of Tim3 on CD4+ or CD8+ T-cells over time or with treatment (Fig. 3d).

### Characterization of human B cells and myeloid cells

**Fig. 4:**
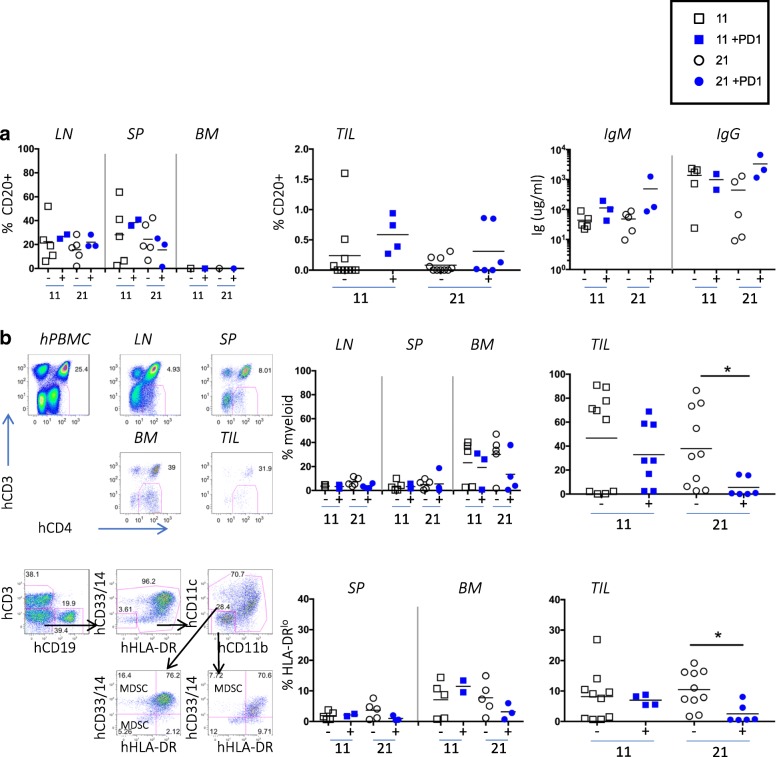
Few B-cells and many myeloid human cells in TILs of TNBC-engrafted hu-CB-BRGS mice*.* **a**, No change observed in human B-cell frequencies (left, middle panels) or function (hIgM and IgG concentrations in serum, right panel) in tumor-bearing, humanized mice with nivolumab treatment. **b**, Decreased human myeloid cell frequencies in TNBC tumors treated with nivolumab. The upper plots depict the flow cytometry gating strategy for identification of the CD4 + CD3- myeloid population among hCD45+ cells from hPBMC (staining control), and indicated organs of a humanized mouse engrafted with TNBC cell line and data from each mouse is shown in upper right graphs. The lower plots show flow cytometry gating (left) for myeloid populations. The myeloid cells are also identified as hCD45+, CD3-, CD19-, (CD14+, CD33+, CD11b + or CD11c+) and the frequency of the HLA-DR^lo/−^ population among these cells is shown. Each dot represents data from the indicated organ from an individual humanized mouse that was either untreated (−, black) or treated with nivolumab (+, blue) for 11 or 21 days, as indicated. *P*-values: * < 0.05

Although human B-cells (CD19+, CD20+) were well represented in the LNs and spleens of humanized mice, there were very few in the TILs, and treatment did not affect their representation (Fig. 4a). However, the presence of human IgM and IgG in the sera suggested B-cells are functional in these mice (Fig. 4a).

The human myeloid lineage is not characteristically well-represented in BRGS or NSG mice; however when gating on human CD45+ cells, we found a notable population of CD3- CD20- CD4+ cells of the myeloid lineage, defined as CD33+ CD11b + or CD14+ [10, 32–34]. To determine if nivolumab treatment influenced the myeloid population in the tumor, we stained and enumerated the frequency of human myeloid cells from the tumor-bearing mice. The frequency of these cells was low in LN and spleens, but they represented up to 40% of the human cells in BM and 80% of the human cells in the TILs. Their frequencies did not change over time or with treatment in the BM; however, they decreased in the TILs after 21 days of treatment (Fig. 4b). We further characterized the CD11b + CD14 + CD33 + CD11c + myeloid populations for expression of HLA-DR, the Major Histocompatibility Complex (MHC) class II molecule that is downregulated on certain subsets of myeloid cells, including monocytic and early myeloid-derived suppressor cells, that are often associated with tumor suppressive phenotype [32, 35]. We found a decrease in the frequency of HLA-DR^lo^ myeloid cells in the TILs after treatment (Fig. 4b).

### Tumor cells

**Fig. 5:**
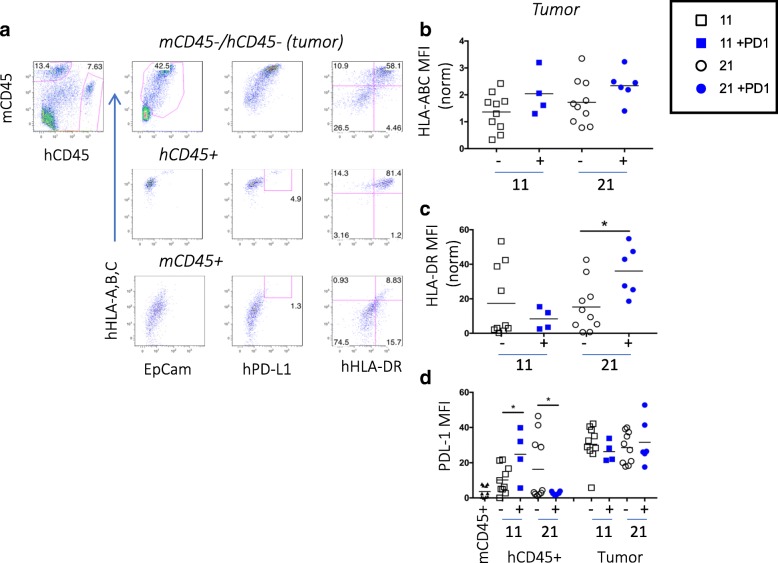
Human PD-L1, MHC class I and class II expression on MDA-MB-231 tumors excised from hu-CB-BRGS. **a**, Representative flow cytometric analyses for expression of HLA-A,B,C (MHC class I), HLA-DR (MHC class II) and hPD-L1 in digested tumor cell suspensions. The tumor cells are defined as mCD45-, hCD45-, HLA-A,B,C^+^, Epcam^+^. The mouse (mCD45) and human (hCD45) hematopoietic cells serve as negative and positive control, respectively. The MFI of HLA-A,B,C (**b**), HLA-DR (**c**) and PD-L1 (**d**) staining is plotted for each tumor. Expression of HLA molecules is normalized to the positive control stained at the same time. Each dot represents data from a tumor from a humanized mouse that was either untreated (−, black) or treated with nivolumab (+, blue) for 11 or 21 days, as indicated. P-values: * < 0.05

In addition to altering immune cells, immunotherapy drugs may also influence the cancer cells or tumor microenvironment (TME), changes that may be vital in the drug’s mechanism of action. Thus, we determined the expression of hPD-L1, HLA-A,B,C (MHC Class I) and HLA-DR (MHC Class II) on TNBC tumor cells using flow cytometry. To distinguish the human tumor cells, we gated out the human and mouse CD45+ cells and analyzed the non-hematopoietic cells expressing HLA-A,B,C and EpCam (Fig. 5a). HLA-DR, but not HLA-A,B,C, expression was higher on the anti-PD-1 treated tumors at day 21 than controls, suggesting nivolumab treatment may improve antigen recognition (Fig. 5b and c). The human hematopoietic cells also expressed these molecules (Fig. 5a). No differences were observed in PD-L1 expression on the tumor cells over time or with treatment; however, there was a significant increase at day 11 followed by a decrease in PD-L1 expression at day 21 on the human CD45+ cells in the TILs from treated mice (Fig. 5d).

### Rational combination therapy reduces tumor growth in the TNBC hu-CB-BRGS model

**Fig. 6:**
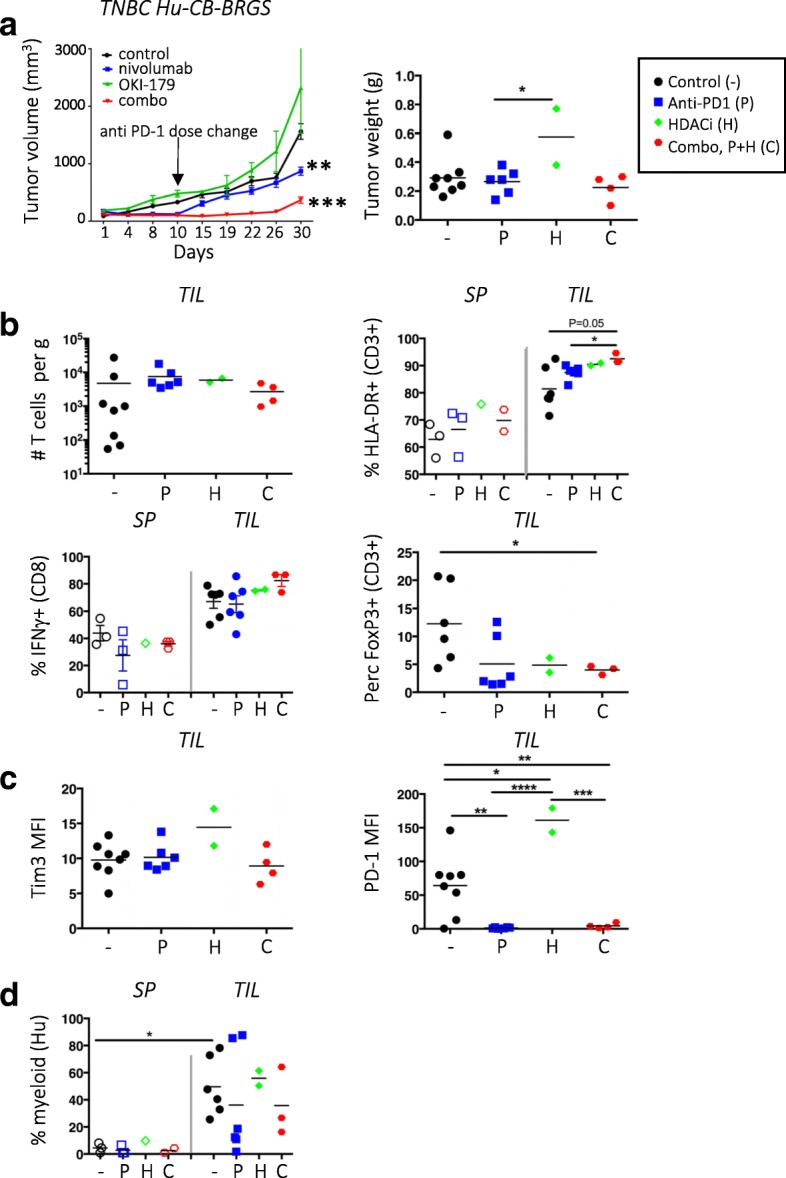
Combination therapy of HDAC inhibitor and nivolumab slows growth of TNBC xenograft in hu-CB-BRGS mice. **a**, Tumor growth curves of MDA-MB-231 TNBC xenografts implanted into hu-CB-BRGS mice that were treated with either control (black), nivolumab (blue), OKI-179 HDACi alone (green), or combination (red). Tumor weights from end of study mice at d30 harvest (control-4 mice/8 tumors; nivolumab-3 mice/6 tumors, OKI-179-1 mouse/2 tumors, combo-2 mice/4 tumors). **b**, T-cell (hCD45 + CD3+) number (top left), frequency of HLA-DR+ of CD3+ (top right), IFNγ+ of CD8+ (bottom left) and FoxP3+ of CD3+ (bottom right) in TILs or lymph organs of TNBC-implanted humanized mice. **c,** MFI of Tim3 (left) or PD-1 (right) on T-cells from TILs. **d**, Frequency of myeloid cells among the hCD45+ population in increased in TILs relative to spleens. Each point represents data from the indicated organ from an individual humanized mouse that was either untreated (−,black), treated with nivolumab (P, blue), OKI-179 (H, green) or both (C, red). P-values: * < 0.05, ** < 0.01, *** < 0.001, **** < 0.0001

HDAC inhibitors (HDACi) have been shown to increase tumor immunogeneicity and improve anti-tumor immune responses in several cancers, including CRC models [36–41]. Thus, we queried whether a Class I selective HDACi drug (OKI-179) would augment the anti-tumor response in combination with nivolumab in the TNBC hu-CB-BRGS model. Interestingly, the combination of OKI-179 and nivolumab significantly inhibited tumor growth (TGI = 77%) more than nivolumab alone (TGI = 45%, Fig. 6a) as also seen in the individual tumor growth curves (Additional file 4: Figure S6a). At day 10, the dose of nivolumab was reduced to allow for detection of combination effects. Immunological phenotyping did not demonstrate enhanced human or T-cell numbers in the TILs (Fig. 6b), although samples were collected at a later timepoint (d30) to allow detection of tumor growth differences and thus may have affected immune comparisons. However, more T-cells were activated (HLA-DR+) in the TILs of mice that received dual therapy and the TILs secreted more IFNγ relative to T-cells from the spleen (Fig. 6b). Also, the percentage of Tregs significantly decreased in the combo-treated mice (Fig. 6b). As above, there was no change in Tim3 expression. PD-1 expression was downregulated with nivolumab treatment, but upregulated in the TILs of OKI-179 single-drug treatment (Fig. 6c). There were also more myeloid cells, including those with HLA-DR^lo^ expression, in the tumor relative to the BM and spleen (Fig. 6d and data not shown). Analysis of the tumors cells showed no change in PD-L1 expression among any treatment groups, suggesting HDAC inhibition does not affect the expression of this inhibitory molecule (Additional file 4: Figure S6b).

### Treatment with anti-PD-1 inhibited growth of a CRC MSI-H PDX that correlated with increased T-cell infiltration in hu-CB-BRGS mice relative to untreated MSI-H or a treated CRC MSS PDX

**Fig. 7:**
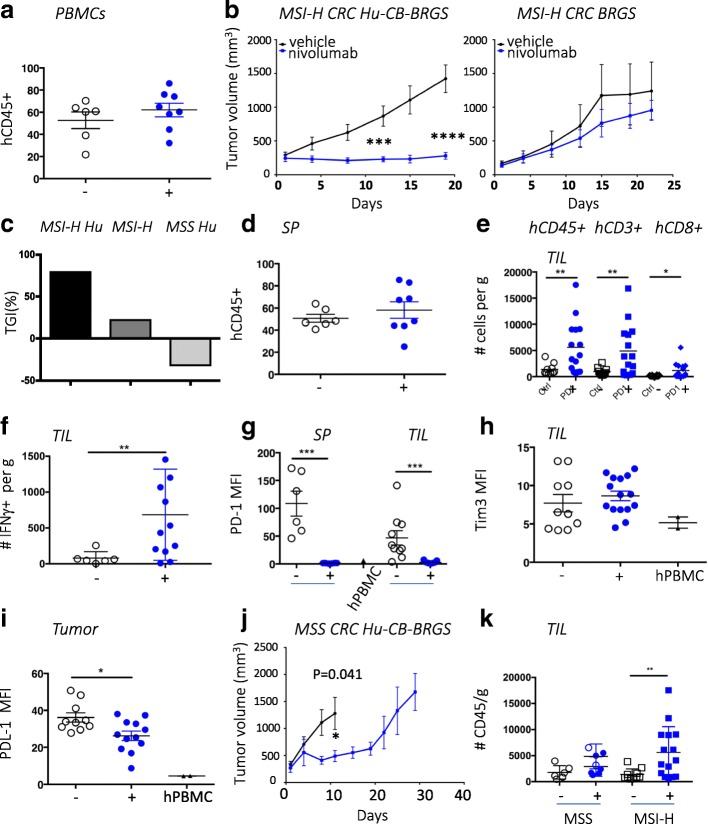
Nivolumab inhibits growth of a MSI-H CRC PDX that correlates with increased human T cells in the tumor compared to the untreated MSI-H CRC PDX or a less-responsive MSS CRC-PDX in hu-CB-BRGS mice. **a,** Human hematopoietic (hCD45+) chimerism in the blood prior to CRC MSI-H PDX implantation. **b**, Tumor growth curves of MDA-C099–203 CRC-PDX (MSI-H) implanted into humanized (left) or non-humanized (right) BRGS mice. Hu-CB-BRGS control group: *n* = 6 mice/10 tumors; nivolumab group: *n* = 8 mice/15 tumors. BRGS control group: *n* = 2 mice/3 tumors; nivolumab group: 3 mice/6 tumors. **c,** Tumor growth inhibition comparison in the hu-CB-BRGS MSI-H, BRGS MSI-H and hu-CB-BRGS MSS mice. **d**, Human hematopoietic (hCD45+) chimerism in the spleen of CRC-MSI-H bearing hu-CB-BRGS mice. **e**-**i**, Immune measurements, as assessed using flow cytometry, of TILs or spleen from CRC MSI-H implanted humanized mice treated (+) or not (−) with nivolumab. **e**, The number of human (hCD45+), T (hCD3+) and CD8 (CD3 + CD8+) T-cells in the TILs. **f**, The numbers of IFNγ-producing T cells in the TILs (gate: hCD45 + CD3+). The MFI of inhibitory receptors PD-1 (**g**) and Tim3 (**h**) on human T cells (hCD45 + CD3+). Human PBMCs served as control. **i,** The MFIs of PD-L1 expression on the CRC MSI-H PDX tumors excised from the humanized mice were determined on the hCD45-mCD45-Epcam+HLA-A,B,C+ population in the TILs. **j**, Tumor growth curves of CRC172 MSS PDX in hu-CB-BRGS mice (*n* = 4 mice/8 tumors per group). **k**, The number of hCD45+ cells in the TILs of either the MSS CRC172 PDX or the MSI-H MDA-C099–203 PDX grown in the hu-CB-BRGS. Open circles in MSS nivolumab treated mice represent tumors from the nivolumab-treated hu-CB-BRGS mouse harvested d10 after start of treatment, with the control mice, while closed blue circles are from the 3 treated hu-CB-BRGS MSS mice harvested on d30. All MSI-H bearing hu-CB-BRGS mice were harvested d23–24 after start of nivolumab. Each point represents data from the indicated organ from an individual hu-CB-BRGS mouse that was either untreated (−, black) or treated with nivolumab (+, blue), as indicated. P-values: * < 0.05, ** < 0.01, *** < 0.001, **** < 0.0001

After recapitulating anti-tumor activity and characterizing immune responses of a TNBC cell line in the hu-CB-BRGS mice, we next sought to determine whether more heterogenous models such as PDXs could be used to evaluate targeted agents in CRC models. To mimic clinical results, we selected MSI-H and MSS CRC models and hypothesized they would be responsive or resistant, respectively, to anti-PD-1 therapy. As above, hu-CB-BRGS mice were bled and stratified into control or treatment arms according to chimerism prior to tumor implantation (Fig. 7a). We established CRC MSS and MSI-H PDX humanized mice, with tumor takes of 94 and 89% respectively. Tumor-implanted animals were then treated with nivolumab using the protocol developed for the TNBC-implanted mice. Significant tumor growth inhibition was observed in treated mice with MSI-H CRC hu-CB-BRGS compared to the control mice (Fig. 7b, left). Nivolumab treatment of the same xenograft implanted into non-humanized BRGS mice did not affect tumor growth (Fig. 7b), whereas overall, TGI was 80% in hu-CB-BRGS versus 23% in non-humanized mice (Fig. 7c).

As in the TNBC experiments, we evaluated the HIS in lymph and tumor tissues from hu-CB-BRGS mice with the PDX in terms of overall human chimerism and immune subsets. We found that the human chimerism in the blood prior to tumor implantation was maintained in the spleen weeks later (Fig. 7d). While we observed no human immune cells in tumors implanted in non-humanized BRGS mice, we identified more immune cells, T-cells, and specifically CD8 T-cells in the anti-PD-1-treated tumors of hu-CB-BRGS mice (Fig. 7e). Moreover, there were significantly more IFNγ-producing human CD8+ TILs in the treated mice (Fig. 7f). Examination of inhibitory receptors demonstrated decreased PD-1 expression in T-cells throughout the body while no changes were observed in expression of Tim3 (Fig. 7g and h). In this model, the tumors exhibited decreased PD-L1 expression with treatment (Fig. 7i). We found no differences in the HIS of non-tumor bearing mice generated from the same CB, with or without nivolumab treatment (data not shown).

A MSS CRC humanized PDX model (CRC172) was similarly treated with nivolumab. Interestingly, an initial inhibition of tumor growth (TGI 92%) was observed in the first 10 days of treatment with nivolumab, followed by a rapid tumor progression (TGI day 30 of − 33%) (Fig. 7j). Progressive tumor growth was observed, similar to what has been reported clinically in MSS CRC patients treated with anti-PD-1 therapy (Fig. 7c) [9]. Interestingly, the number of human TILs was significantly increased in the MSI-H tumors, but not the MSS tumors, of nivolumab-treated hu-CB-BRGS mice (Fig. 7k). In the MSS CRC model, we detected no significant differences in T-cell activation, functional markers and inhibitory receptors between vehicle control and nivolumab-treated mice, albeit there were very few T-cell infiltrates to properly analyze at end of study (data not shown).

Using multispectral IHC, we examined the human immune infiltrates in the MSI-H and MSS tumor samples to garner information about cellular location. This analysis supported the flow cytometry data with clear populations of T-cells in the nivolumab-treated CRC MSI-H PDX but very few if any found in the untreated MSI-H or in any CRC MSS PDX tumor or adjacent stroma that had grown in the humanized mice (Additional file 4: Figure S7). Our results with low immune responses to MSS CRC confirm the clinical data [9] and emphasize the need to investigate combinations that may increase the immunogenicity, and perhaps the accessibility, of these tumors.

## Discussion and conclusions

Currently, there is a well-recognized need to improve immunologic responses to tumors, to understand which tumors are responsive to immunotherapy, and the mechanisms by which they respond. Given the cost and complexities associated with clinical trials, the disadvantages of in vitro models and poor track record of syngeneic mouse models to predict responses in humans, there is a clear need for an in vivo small animal model for the testing of rational combination strategies to enhance the efficacy of immunotherapy [42].

Here we provide evidence that hu-CB-BRGS mice are suitable to study in vivo responses of human leukocytes to human tumors. With years of humanized mice experience as a guideline, our team developed and characterized a novel human immune-oncology model using BALB/c-Rag2^null^Il2rγ^null^SIRPα^NOD^ (BRGS) hosts. Similar to the studies in NSG mice [23], we found that our mice can be implanted with cell line or PDX tumors with a take-rate > 90%. However, in our studies, we waited longer to implant tumors, upon confirmation of T lymphocytes among the human CD45+ cells in the blood, a factor that correlates with LN development. Futhermore, we also allowed tumors to grow to a larger size before treatment, to simulate a more realistitc clinical scenario. Notably, between 10 and 50 humanized mice can be generated from a single CB, allowing multiple treatment groups per experiment and statistical robustness. In our model, we observed reproducible responses to nivolumab using the MDA-MB-231 cell line in four separate experiments with four distinct CB donors (Figs. 1, 2 and 6 and data not shown), and we further demonstrated an enhanced response using an HDACi as combination therapy. In addition, anti-PD-1 therapy reduced tumor growth in a CRC MSI-H that was maintained more than in a CRC MSS PDX model, somewhat similar to what is reported in human patients [9, 43]. Since the CRC MSI-H PDX was derived from a primary tumor while the CRC MSS from a metastatic, we cannot rule out this difference might also play a role in the differential immune response; moreover, we are aware of the possible differences and limitations existing in the evaluation of tumor responses to immunotherapies between an orthotopic versus a sub-cutaneous model, but the literature does not currently report evidence of superiority of either model. However, we have the ability to further investigate this question in our model.

The ability of the humanized mice to accept and support allogeneic tumor growth, non-HLA matched with the CB donor used to humanize the mouse, strongly suggests the HIS in these mice is immunosuppressed, and thus may recapitulate some aspects of the TME. T-cells in the humanized mice express high levels of PD-1, and treatment of mice with anti-human PD-1 therapy results in tumor-specific responses. Therefore, the HIS is not incapable of rejecting the tumor, but requires modulation to mount an effective immune response. We view the immunosuppressed nature of the HIS in these mice as a quality necessary for the acceptance of the tumors and an important basis for the utility of this novel preclinical platform. In essence, these experiments measure the degree to which we alleviate this immunosuppression, which we quantify both by tumor growth measurements and changes in molecular readouts of the immune system and the tumor.

In our humanized mouse model, the LNs, spleen, and tumor tissues are readily accessible, enabling in-depth interrogation of the immune response in both peripheral lymph and tumor. In these studies, we demonstrated an increase in human cells, most notably T-cells, in the TILs correlates with successful nivolumab treatment, as has been observed in TILS from patients analyzed prior to treatment [44, 45]. We verified that the human T-cells are in fact derived from CB hematopoietic stem cells and are not passenger leukocytes from the implanted tumor, as no hCD45^+^ cells are detected in tumors explanted from non-humanized BRGS mice. The lack of human (and T-cell) infiltration into the CRC MSS PDX tumors may explain its reduced response to nivolumab compared to the CRC MSI-H PDX model in which significantly larger numbers of human T-cells (including CD8^+^ IFNγ^+^) correlated with its tumor growth inhibition upon anti-PD-1 therapy.

T-cells (and all immune cell subsets) can be either inflammatory or suppressive and therefore the presence of immune subsets alone does not indicate tumor-destructive responses. For this reason, we also examined T-cell activation (HLA-DR and CD69 upregulation), cytotoxicity-associated protein production (IFNγ or GrB) and suppressor cell transcription factor expression (FoxP3) among the CD4 and CD8 subsets. In our representative TNBC hu-CB-BRGS model, the tumors treated with the anti-PD-1 therapy had an increase in T-cell activation frequency of GrB earlier and IFNγ secretion later, confirming that the T-cells present in the humanized mice were functional and cytotoxic, capable of inducing tumor cell death. We observed significant populations of both CD4+ and CD8+ cytotoxic GrB+ and IFNγ+ cells, as well as regulatory FoxP3+ cells. Although a higher percentage of CD8+ T-cells were cytotoxic, and conversely more of the regulatory T-cells were CD4+, the overlap of functions among the subsets illustrates the plasticity of our HIS.

Currently, there is intense interest in understanding productive immune responses to checkpoint inhibition, with an explosion of high impact publications in recent months [30, 44–47]. Using “unbiased”, approaches, these studies concluded that “reinvigorated” CD8+ T cells, along with decreased Tregs in a subset of these reports, showed the greatest correlation to anti-PD-1 therapy in patients' responses, consistent with our studies using the TNBC cell line or CRC PDX models. Our data also suggest the presence of CD8 + IFNγ+ T cells relative to the tumor burden (per gram) is the most relevant measurement correlating with success in both the TNBC and CRC PDX models. In our model, we have the ability to alter the treatment timecourse starts according to tumor size, and to evaluate the effect of tumor burden, shown to be a negative prognostic factor in patient studies, on checkpoint blockade therapy [30, 48]. Although some of these studies analyzed the TILs themselves [45, 47], others were restricted to characterizing immune cells in patient blood, in which significant differences were only observed at a single timepoint [30]. Importantly, these human studies are restricted to limited patient tumor samples and tumor types, and are furthermore very expensive and time-consuming. Given the vast heterogeneity of tumors and evolving immune responses, it is challenging to identify notable mechanisms among the highly variable datasets. In comparison, our model is a relatively cost and time effective alternative to interrogate immune responses to a particular tumor using multiple mice with genetically-identical immune systems, thus reducing inherent variability.

In contrast to reports studying immune responses to anti-PD-1 in human melanoma patients we observed no compensatory changes in expression of inhibitory receptors on human leukocytes, including Tim3 as we have shown in this study or Lag3 or CTLA4 in other studies**.** However, the higher expression of Tim3 in our TILs relative to peripheral lymph is consistent with reports of increased exhausted T cells in tumors. Immune responses to tumors may indeed be quite heterogeneous, tumor and time dependent, and although reinvigorated T cells play a significant role, other studies have shown a role for CD4+ T cells [49], non-exhausted CD8+ T cells, as well as decreased Tregs and myeloid cells [50].

An added advantage of the hu-CB-BRGS model is that we detect considerable myeloid infiltration in the tumors at early timepoints and without treatment, which was significantly reversed with nivolumab administration over 21 days, consistent with increased T-cell presence. This finding indicates two important considerations: 1) that this humanized mouse model supports myeloid populations in significant frequencies in the physiologically relevant BM that are capable of homing to the tumor, and 2) the myeloid population is the dominant population in the TILs of untreated TNBC MDA-MB-231 tumors, as well as other PDXs we have studied (data not shown), and consistent with findings in many human and mouse tumors [32]. Furthermore we observed diminished myeloid populations, including those of the HLA-DR^lo^ “suppressive” phenotype, in anti-PD-1 treated hu-CB-BRGS mice in the TNBC model, consistent with the increased T cell/myeloid ratio observed in successful immune responses [35, 50].

Similar to studies examining immune responses to anti-PD-1 in mouse syngeneic models [47], our studies also found that differences observed among human leukocytes from either untreated or nivolumab-treated humanized mice were restricted to the TILs and not significant in other lymph tissue (LNs, spleen, BM), although these organs contained T-cells with high levels of PD-1 expression, and thus should be targets of the drug. This central finding is vital to the utility of this model and suggests that the therapies are impacting tumor-specific immune cells more than other self-antigen specific cells. It also implies that analysis of immunotherapies are best examined in the tumor tissue and that other lymph organs, i.e. peripheral blood, are not indicative of tumor-specific immune responses.

The Hu-CB-BRGS model has limitations, such as limited NK cells, migration of B-cells to the tumor (a similar lack of B-cells were observed in HuNSG PDX models) and the HLA-mismatch of tumor and CB-derived immune system [23]. For this latter issue, even if we were able to match the HLA of the tumor and CB, the T-cells would still be selected on mouse thymus, and therefore, would remain in essence “xeno” to the tumor. The mis-matched T-cell selection is likely responsible for the immunosuppressed environment within the humanized mouse. Unless we have a complete thymus-blood-tumor match, the likelihood of a tumor growing in the mouse is predicted to decrease with increased matching, and indeed others have shown that the degree of HLA-matching in a PDX hu-NSG model does not increase response to immunotherapy [23]. This challenge is currently being addressed by our lab and other groups. We must also consider the chimerism inherent in this model and work is also being done to address the role of mouse monocytes on the anti-tumor response.

The immune system-tumor response is a dynamic process that can change over time and with treatment. In humanized mouse models, the development of T-cells can take months and have mouse-to-mouse, or more frequently, CB-to-CB variability [20]. It is thus important to consider these kinetics for timing of tumor implantation. In addition, the TME can evolve over time, and these changes can dictate immune phenotypes present in the tumor. The timecourse experiment indicated differences in the immune system at 11 days post treatment relative to 21 days. However, tumor regrowth can also occur later, likely due to increased immunosuppression in the tumor. Thus the need to gather data for a growth curve should be balanced against evaluation of the immune system in a reasonable timeframe. Our analysis of the HIS revealed enhanced cytotoxic, inflammatory T-cells in the tumors of nivolumab-treated mice, although there were similar responses in some untreated tumors and variability among treated tumors. This likely reflects the ongoing interplay between active immune responses and immunosuppression in this model. Considering the dynamic nature of the response as well as the variability of human chimerism in this model, the differences we observed among several biologically-relevant immune parameters provides strong evidence for the validity of this model.

The specificity of the T-cell response to the tumor is still unclear, yet most likely includes allo (human), xeno (mouse stroma) and tumor specificities. Studies are being conducted to further answer this important question. However, the reduced tumor growth with checkpoint inhibition, in the absence of major autoimmune side-effects in the mice or GVHD, supports a tumor-specific response. The lack of autoimmunity is likely a difference in T-cell specificities available to the tumor relative to self-antigens, which would have lower affinities due to intact central tolerance. One would predict the responses to the tumor would be more robust in these mice, which should harbor a higher frequency of tumor-reactive specificities, than a patient, but alterations in TME responsible for the immunosuppression should be similar. Therefore, this model should theoretically stratify best combination therapies by measuring the release of immunosuppression, specifically to a foreign graft, with tumor growth as a readout. The tumor type, and likely its degree of immunosuppression as well as tumor-bearing antigens, would influence the ability of the immune system to respond, and indeed we found differences in the responses to two different CRC PDXs. More importantly, we demonstrated that the model offers the ability to study combination therapies, which are often designed to alter the immunosuppressive environment of the tumor. Further investigations are needed to identify the particular mechanism of action of the HDACi drug, whether it is affecting the TME itself or also the immune cells. These investigations are feasible with this model. Furthermore, this model offers the flexibility to test kinetics of drug dosing (i.e. does the drug upregulate HLA molecules and thus should precede checkpoint inhibition?). Overall our data show that the hu-CB-BRGS model is ideally-suited to test changes in tumor immunogenicity using different cancer cells and treatments, and to analyze their effect on a human immune response with data from multiple mice, prior to clinical trials. Perhaps the greatest value of this complex model will be the ability to utilize it not only in prioritizing rational combinations among the infinite available, but to also develop mechanistic immune-response hypotheses which then can be tested in the clinic within the context of clinical efficacy.

## Additional files

Additional file 1: Supplementary Methods. (DOCX 26 kb)

Additional file 2: **Table S1.** Experimental details and timeline for tumor growth studies in BRGS and hu-CB-BRGS mice. (DOCX 20 kb)

Additional file 3: **Table S2.** Flow Cytometric Abs and Reagents. (XLSX 49 kb)

Additional file 4: **Figure S1.** Experimental timeline for generation of PDX/cell line-implanted hu-CB-BRGS mice. **Figure S2.** Expression of hPD-L1 on MDA-MB-231 TNBC cell line. MDA-MB-231 cells grown in culture were collected and stained for expression of human PD-L1. **Figure S3.** Human chimerism and cell numbers in TNBC MDA-MB-231 cell line implanted hu-CB-BRGS mice harvested at d11 and d21 post treatment. **a,** Equivalent human hematopoietic (left), T (middle, gate: hCD45+) and CD8 (right, gate: hCD45+,CD3+) chimerism in blood of humanized mice among experimental (d11 or d21 harvest, control (−) or anti-PD-1 treatment (+) groups. **b,** Human hematopoietic (hCD45+) and T (CD3+) cell numbers in lymph organs of TNBC-bearing hu-CB-BRGS mice at harvest. **Figure S4.** Immunohistochemistry analysis of human and mouse chimerism in TNBC MDA-MB-231 cell line implanted hu-CB-BRGS mice. **a,** Representative IHC slides from untreated and nivolumab-treated MDA-MB-231 tumors explanted from hu-CB-BRGS mice 11 or 21 days after start of treatment. **b,** Increased human T-cell (CD3) densities in tumors of hu-CB-BRGS mice treated with nivolumab for 21 days. **Figure S5.** Expression of CD25 (clone M-A251) on FoxP3+ CD4+ and CD8+ T cells (hCD45 + CD3+) in LN and spleens of hu-CB-BRGS mice. **a**, Representative flow cytometry staining and **b**, cumulative data showing percentage of FoxP3+ T cells (left) and percentage of CD25+ among the FoxP3+ T cells (right). **Figure S6.** Individual data points and expression of hPD-L1 on MDA-MB-231 TNBC cell line harvested from hu-CB-BRGS mice**. a,** Tumor growth curves of untreated (black), nivolumab-treated (red), OKI-179-treated (green) and combination (red) of the TNBC hu-CB-BRGS mice. **b,** Tumors were identified as mCD45-, hCD45-, Epcam+ or HLA-A,B,C+. **Figure S7.** Increased detection of human T cells in IHC sections from nivolumab-treated MSI-H PDX relative to untreated MSI-H PDX or nivolumab-treated MSS PDX. (PPTX 16200 kb)

## Abbreviations

Ab: Antibody
BM: Bone marrow
BRGS: BALB/c-Rag2^null^Il2rγ^null^SIRPα^NOD^
CB: Cord blood
CRC: Colorectal cancer
CTLA-4: Cytotoxic T-lymphocyte-associated protein 4
CyTOF: Cytometer Time-of-Flight
DAPI: 4′,6-diamidino-2-phenylindole
ELISA: Enzyme-linked immunosorbent assay
EpCam: Epithelial cell adhesive molecule
FBS: Fetal bovin serum
FoxP3: Forkhead box P3
GrB: Granzyme B
HDAC: Histone deacetylase
HIS: Human immune system
HLA: Human Leukocyte Antigen
hu-CB-BRGS: Humanized (from) cord blood BRGS
IFNγ: Interferon gamma
Ig: Immunoglobulin
IHC: Immunohistochemistry
LN: Lymph node
MHC: Major Histocompatibility Complex
MSI: Microsatellite Instability
MSS: Microsatellite Stable
NK: Natural killer
NSG: NOD-SCID-IL2RγC^null^
PBMC: Peripheral blood mononuclear cell
PD-1: Programmed Cell Death Protein-1
PD-L1: Programmed Cell Death Protein-1 ligand
PDX: Patient-Derived Xenograft
RPMI: Roswell Park Memorial Institute media
SEM: Standard error of the mean
TGI: Tumor growth inhibition
TILs: Tumor infiltrating leukocytes
TIM-3: T-cell immunoglobulin and mucin-domain containing-3
TME: Tumor microenvironment
TNBC: Triple negative breast cancer
UCD-AMC: University of Colorado Denver-Anschutz Medical Center
