# Supplementary material for: Characterization of immune responses to anti-PD-1 mono and combination immunotherapy in hematopoietic humanized mice implanted with tumor xenografts
Source: J Immunother Cancer. 2019 Feb 8;7:37. doi: 10.1186/s40425-019-0518-z (PMC6368764; doi:10.1186/s40425-019-0518-z)
Supplement: Supplementary file 1 — Supplementary Methods. (DOCX 26 kb) [file 40425_2019_518_MOESM1_ESM.docx]

Additional File 1

**Supplementary Methods**

## Mice

BALB/c-*Rag2^null^Il2rγ^null^Sirpa^NOD^* (BRGS) recipient mice were bred and engrafted in a biosafety level 2 room at the University of Colorado Denver-Anschutz Medical Center (UCD-AMC) vivarium and maintained on a diet which included septra (Uniprim diet, Harlan) every other week to prevent opportunistic infections [[24](#_ENREF_24), [25](#_ENREF_25)]. Five- to six-week-old female athymic nude (nu/nu) mice (Envigo, formally Harlan Sprague Dawley) were purchased and used for growth of CRC PDX models prior to implantation into BRGS mice. All studies were conducted with prior approval from the University of Colorado Animal Care and Use Committee and in a facility accredited by the American Association for Accreditation of Laboratory Animal Care.

**PDX generation**

Briefly, patients undergoing either removal of a primary CRC (MSI-H, MDA-C0999-203 F7; MSI status verified by PCR in a CLIA-CAP certified lab at MD Anderson) or metastatic tumor (MSS, CRC172 F7 at the University of Colorado Hospital) were consented in accordance with IRB-approved institutional protocols.

**Tumor growth calculations**

The following equation was used to estimate tumor volume: (length × width^2^) × 0.52 and recorded in the Study Director software package (Studylog Systems). Tumor growth curves are presented as average tumor volume±SEM for each treatment group in study. For tumor growth curves, day 0 represents start of treatment, which took place 12 to 15 days after the implantation of tumors.

**Human chimerism determination in blood of hu-CB-BRGS mice**

As described previously, the hu-CB-BRGS mice were bled retro-orbitally and peripheral blood mononuclear cells (PBMCs) were evaluated for mouse and human hematopoietic cell chimerism (mCD45 or hCD45) [[20](#_ENREF_20), [28](#_ENREF_28)]. Human hematopoietic chimerism is calculated as hCD45+/(mCD45+ + hCD45+). Human T-cells (hCD3, hCD8), human B-cells (hCD20) and human PD-1 expression were evaluated twice between 8 and 18 weeks prior to tumor implantation and mice were subsequently sorted into equivalent experimental and control groups bas ed on chimerism.

**Flow cytometric analysis of human immune system in tumor-implanted hu-CB-BRGS**

The following immune-related proteins were analyzed from tissues of hu-CB-BRGS mice: Mouse leukocytes (mouse CD45) or human lymphocytes (human CD45, CD3, CD4, CD8, CD19, CD20), Tregs (CD25, intracellular FoxP3), activated T-cells (CD69, HLA-DR), effector T-cells (intracellular GrB, IFNγ, and Tbet), inhibitory receptors (PD-1, Tim3), myeloid cell subsets (CD33, CD11b, CD11c, HLA-DR) and the immune status of the tumor (HLA-ABC class I, HLA-DR class II, PD-L1). Stained cells were run on a Cyan analyzer (Beckman Coulter) at the UCD Cancer Center Flow Cytometry Shared Resource [[19](#_ENREF_19), [28](#_ENREF_28)]. All samples were initially stained for human, T and B cell chimerism. Subsequent stains were performed on those samples with identifiable populations of interest (e.g. IFNγ and GrB/FoxP3 stain is only included for those samples with human T cells); therefore in some analyes there are fewer samples for some parameters thatn the human T and B cell chimerism analysis. Intracellular staining of proteins was performed with 2% paraformaldehyde fixation followed by saponin permeabilization and staining, as described previously [[28](#_ENREF_28)]. Mice with <5% human chimerism in the spleen were excluded from the study. For analysis of IFNγ production by human T-cells, approximately 2x10^6^ LN cells, splenocytes, or tumor-infiltrating leukocytes (TILs) were stimulated overnight with Cell Stimulation Cocktail in RPMI-10% FCS media (described above) at 37°C in a humidified chamber containing 5% CO_2_. Both unstimulated and stimulated human PBMCs were included in each assay as negative and positive controls, respectively. After 14-16 h, GolgiPlug was added to each well. Four hours later, the cells were washed once with staining buffer, and extracellular staining was performed to identify human CD8 T-cells, followed by intracellular staining for detection of IFNγ and Tbet. Data were analyzed using FlowJo sofware (Tree Star). Cells were gated on single-cells based on forward and side scatter followed by doublet discrimination.

## Vectra 3.0 Multispectral imaging of tumors

Four micron sections of formalin-fixed tumor tissues were mounted onto glass slides and sequentially stained for human CD3, CD33, CD14, CD8, pan-cytokeratin, and mouse CD45 on a Bond RX autostainer (Leica). Briefly, slides were dewaxed (Leica), heat treated in ER2 antigen retrieval buffer for 20 min at 93^o^ C (Leica), blocked in Ab Diluent (Perkin Elmer), incubated for 30 min with the primary Ab, 10 min with horseradish peroxidase (HRP)-conjugated secondary polymer (anti-mouse/anti-rabbit, Perkin Elmer), and 10 min with HRP-reactive OPAL fluorescent reagent (Opal 540, Perkin Elmer). Slides were washed between staining steps with Bond Wash (Leica) and stripped between each round of staining with heat treatment in appropriate antigen retrieval buffer. After the final staining round, the slides were heat-treated in ER1 antigen retrieval buffer, stained with spectral DAPI (Perkin Elmer), and coverslipped with Prolong Diamond mounting media (Thermo Fisher). Whole slide scans were collected using the 10x objective and approximately 10 regions were selected for multispectral imaging with the 20x objective. The multispectral images were analyzed with inForm software (Perkin Elmer) to unmix adjacent fluorochromes, subtract autofluorescence, segment the tissue into tumor regions and stroma, segment the cells into nuclear and membrane compartments, and to phenotype the cells according to morphology and cell marker expression.
