# Supplementary material for: Characterization of immune responses to anti-PD-1 mono and combination immunotherapy in hematopoietic humanized mice implanted with tumor xenografts
Source: J Immunother Cancer. 2019 Feb 8;7:37. doi: 10.1186/s40425-019-0518-z (PMC6368764; doi:10.1186/s40425-019-0518-z)
Supplement: Supplementary file 2 — Table S1. Experimental details and timeline for tumor growth studies in BRGS and hu-CB-BRGS mice. (DOCX 20 kb) [file 40425_2019_518_MOESM2_ESM.docx]

Table S1. Experimental details and timeline for tumor growth studies in BRGS and hu-CB-BRGS mice.

| Tumor | Tumor Growth Curve | Groups | # mice injected with tumor | Age of mouse tumor injection | Treatment start date (post tumor) | Harvest Day (post treatment)  (d) | # mice lost to death | # mice excluded for low chimerism* | # mice final analysis | # tumors final analysis |
| --- | --- | --- | --- | --- | --- | --- | --- | --- | --- | --- |
| MDA-MB-231 | Fig 1c | BRGS Ctrl | 1 |  |  | 34 | 0 | 0 | 1 | 2 |
|  |  | BRGS Nivo | 1 |  | d16 | 34 | 0 | 0 | 1 | 2 |
|  |  | Hu-BRGS Ctrl | 3 | 17 wks |  | 30 (2)  38 (1) | 0 | 0 | 3 | 6 |
|  |  | Hu-BRGS Nivo | 3 |  | d16 | 30 (2)  38 (1) | 0 | 0 | 3 | 6 |
|  |  |  |  |  |  |  |  |  |  |  |
| MDA-MB-231 | Fig 2a | Hu-BRGS Ctrl | 5 | 20-22 wks |  | 11-13 | 0 | 0 | 5 | 10 |
|  |  |  | 5 |  |  | 21-23 | 0 | 0 | 5 | 10 |
|  |  | Hu-BRGS Nivo | 5 |  | d14-15 | 11-13 | 0 | 3 | 2 | 4 |
|  |  |  | 4 |  |  | 21-23 | 0 | 1 | 3 | 6 |
|  |  |  |  |  |  |  |  |  |  |  |
| MDA-MB-231 | Fig 6a | Hu-BRGS Ctrl | 4 | 20-21 wk |  | 30 | 0 | 0 | 4 | 8^2^ |
|  |  | Hu-BRGS Nivo | 4 |  | d10 | 30 | 1 |  | 3 | 6 |
|  |  | Hu-BRGS OKI-179 | 4 |  | d10 | 21 (1)  30 (1) | 3^1^ | 0 | 1 | 2 |
|  |  | Hu-BRGS Combo | 4 |  | d10 | 30 | 2 | 0 | 2 | 4^3^ |

| Tumor | Tumor Growth Curve | Groups | # mice injected with tumor | Age of mouse tumor injection | Treatment start date (post tumor) | Harvest Day (post treatment)  (d) | # mice lost to death | # mice excluded for low chimerism | # mice final analysis | # tumors final analysis |
| --- | --- | --- | --- | --- | --- | --- | --- | --- | --- | --- |
| MDA-C099-203 CRC MSI-H PDX | Fig 7b | BRGS Ctrl | 2 |  |  | 36 (1)  41 (1) | 0 | 0 | 2 | 3 |
|  |  | BRGS Nivo | 3 |  | d15 | 36 (1)  41 (2) | 0 | 0 | 3 | 6 |
|  |  | Hu-BRGS Ctrl | 6 | 17-20 wks |  | 23-24^4^ | 0 | 0 | 6 | 10 |
|  |  | Hu-BRGS Nivo | 8 |  | d30 | 23-24^4^ | 0 | 0 | 8 | 15 |
|  |  |  |  |  |  |  |  |  |  |  |
| CRC172  MSS PDX | Fig 7j | Hu-BRGS Ctrl | 4 | 18-21 wks |  | 10 (4) | 0 | 0 | 4 | 6 |
|  |  | Hu-BRGS Nivo | 4 |  | d12 | 10 (1)  30 (3) | 0 | 0 | 4 | 8 |
|  |  |  |  |  |  |  |  |  |  |  |

^*^Low chimerism is defined as less than 5% human chimerism in both bone marrow and spleen at harvest.

^1^One mouse was found dying and was analyzed for chimerism immediately – no other flow data available for this mouse

^2^ Two tumors had very low T cell chimerism in TIL so not included in further analysis

^3^One tumor had very low T cell chimerism in TIL so not included in further analysis

^4^All mice were taken down in two consecutive days.
