# Supplementary material for: Characterization of immune responses to anti-PD-1 mono and combination immunotherapy in hematopoietic humanized mice implanted with tumor xenografts
Source: J Immunother Cancer. 2019 Feb 8;7:37. doi: 10.1186/s40425-019-0518-z (PMC6368764; doi:10.1186/s40425-019-0518-z)
Supplement: Supplementary file 4 — Figure S1. Experimental timeline for generation of PDX/cell line-implanted hu-CB-BRGS mice. Figure S2. Expression of hPD-L1 on MDA-MB-231 TNBC cell line. MDA-MB-231 cells grown in culture were collected and stained for expression of human PD-L1. Figure S3. Human chimerism and cell numbers in TNBC MDA-MB-231 cell line implanted hu-CB-BRGS mice harvested at d11 and d21 post treatment. a, Equivalent human hematopoietic (left), T (middle, gate: hCD45+) and CD8 (right, gate: hCD45+,CD3+) chimerism in blood of humanized mice among experimental (d11 or d21 harvest, control (−) or anti-PD-1 treatment (+) groups. b, Human hematopoietic (hCD45+) and T (CD3+) cell numbers in lymph organs of TNBC-bearing hu-CB-BRGS mice at harvest. Figure S4. Immunohistochemistry analysis of human and mouse chimerism in TNBC MDA-MB-231 cell line implanted hu-CB-BRGS mice. a, Representative IHC slides from untreated and nivolumab-treated MDA-MB-231 tumors explanted from hu-CB-BRGS mice 11 or 21 days after start of treatment. b, Increased human T-cell (CD3) densities in tumors of hu-CB-BRGS mice treated with nivolumab for 21 days. Figure S5. Expression of CD25 (clone M-A251) on FoxP3+ CD4+ and CD8+ T cells (hCD45 + CD3+) in LN and spleens of hu-CB-BRGS mice. a, Representative flow cytometry staining and b, cumulative data showing percentage of FoxP3+ T cells (left) and percentage of CD25+ among the FoxP3+ T cells (right). Figure S6. Individual data points and expression of hPD-L1 on MDA-MB-231 TNBC cell line harvested from hu-CB-BRGS mice. a, Tumor growth curves of untreated (black), nivolumab-treated (red), OKI-179-treated (green) and combination (red) of the TNBC hu-CB-BRGS mice. b, Tumors were identified as mCD45-, hCD45-, Epcam+ or HLA-A,B,C+. Figure S7. Increased detection of human T cells in IHC sections from nivolumab-treated MSI-H PDX relative to untreated MSI-H PDX or nivolumab-treated MSS PDX. (PPTX 16200 kb) [file 40425_2019_518_MOESM4_ESM.pptx]

## Slide 1
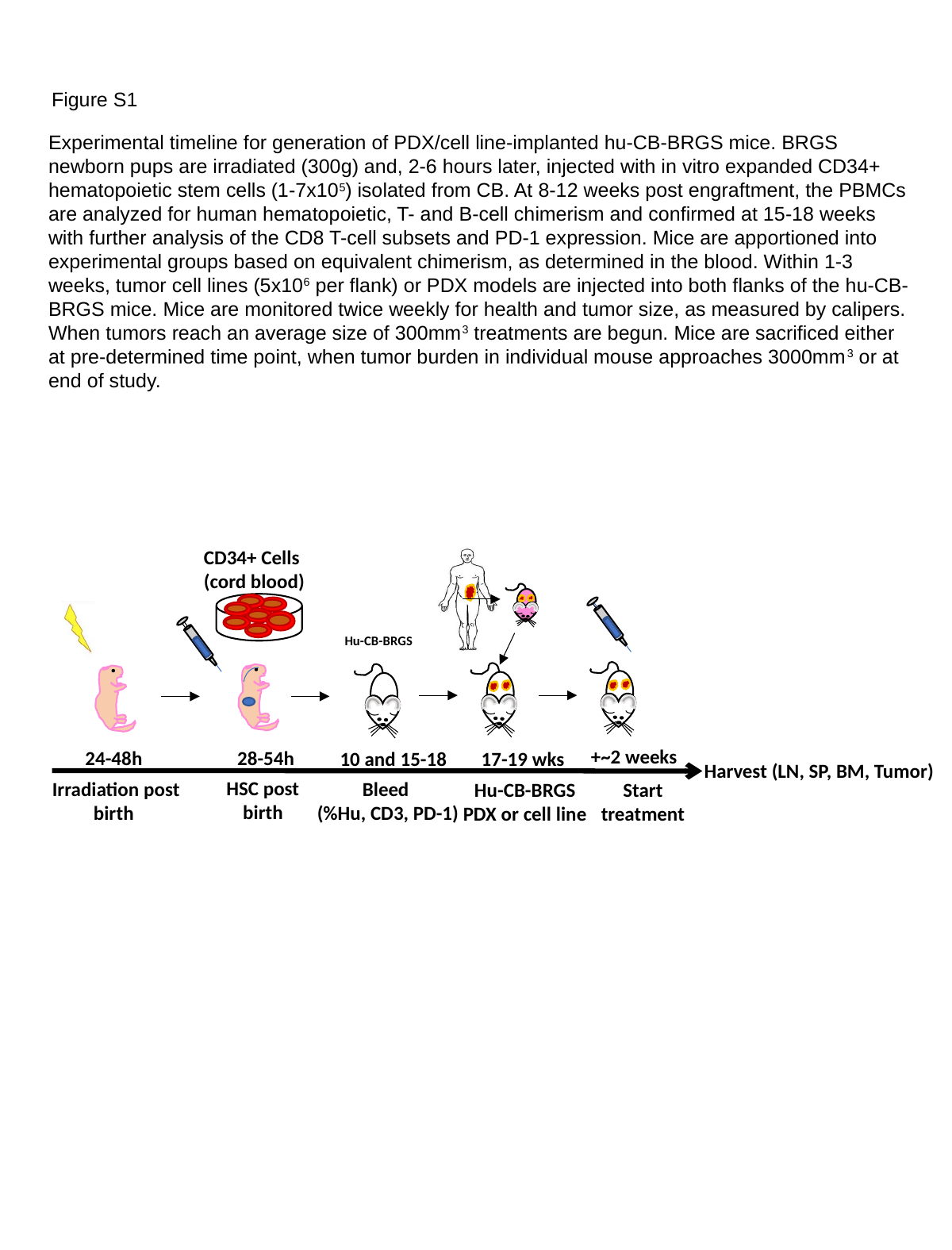

Figure S1
Experimental timeline for generation of PDX/cell line-implanted hu-CB-BRGS mice. BRGS newborn pups are irradiated (300g) and, 2-6 hours later, injected with in vitro expanded CD34+ hematopoietic stem cells (1-7x10^5^) isolated from CB. At 8-12 weeks post engraftment, the PBMCs are analyzed for human hematopoietic, T- and B-cell chimerism and confirmed at 15-18 weeks with further analysis of the CD8 T-cell subsets and PD-1 expression. Mice are apportioned into experimental groups based on equivalent chimerism, as determined in the blood. Within 1-3 weeks, tumor cell lines (5x10^6^ per flank) or PDX models are injected into both flanks of the hu-CB-BRGS mice. Mice are monitored twice weekly for health and tumor size, as measured by calipers. When tumors reach an average size of 300mm3 treatments are begun. Mice are sacrificed either at pre-determined time point, when tumor burden in individual mouse approaches 3000mm3 or at end of study.
CD34+ Cells
(cord blood)
Hu-CB-BRGS
+~2 weeks
24-48h
28-54h
17-19 wks
10 and 15-18
Harvest (LN, SP, BM, Tumor)
HSC post birth
Bleed
(%Hu, CD3, PD-1)
Irradiation post birth
Start treatment
Hu-CB-BRGS PDX or cell line

## Slide 2
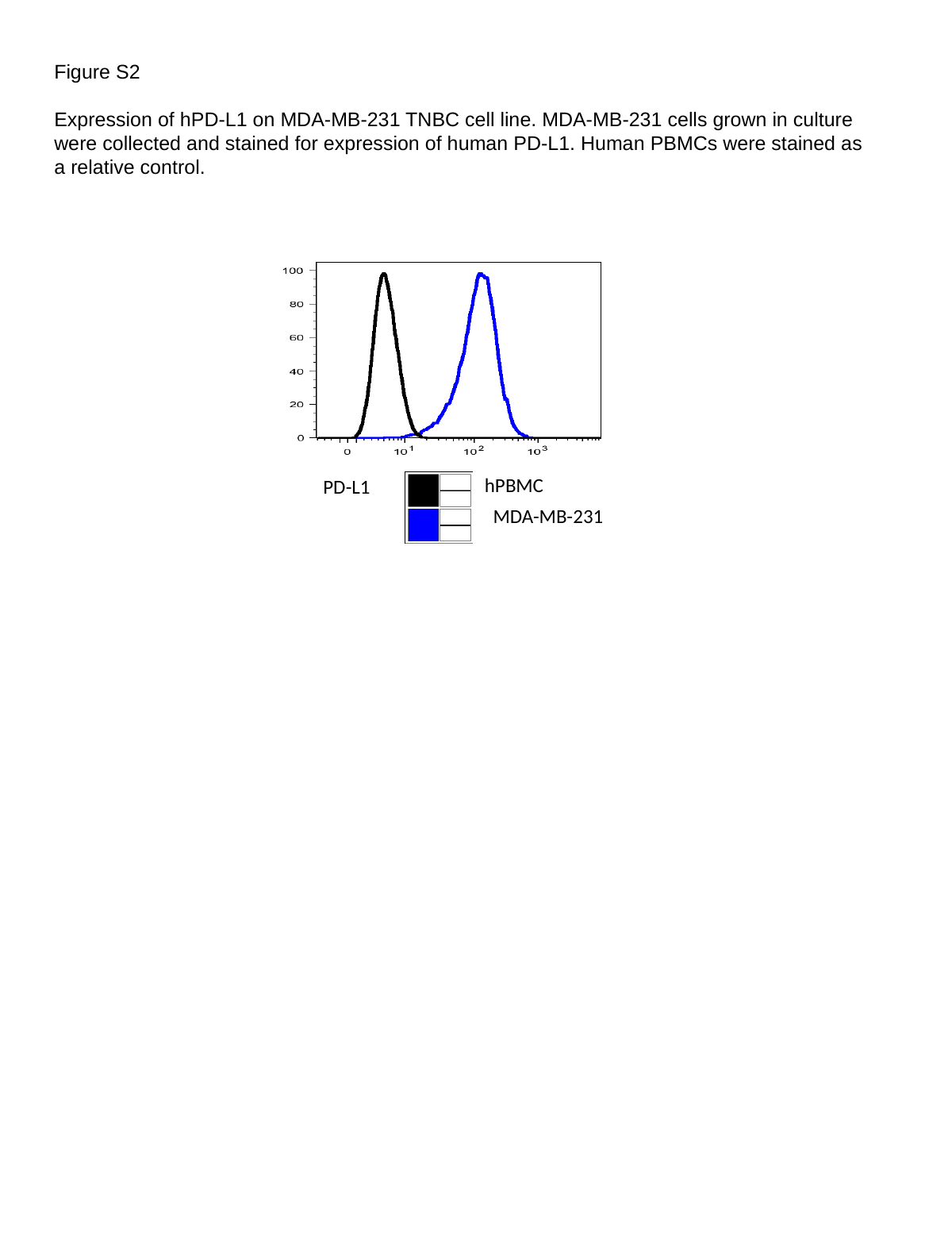

Figure S2
Expression of hPD-L1 on MDA-MB-231 TNBC cell line. MDA-MB-231 cells grown in culture were collected and stained for expression of human PD-L1. Human PBMCs were stained as a relative control.
hPBMC
PD-L1
MDA-MB-231

## Slide 3
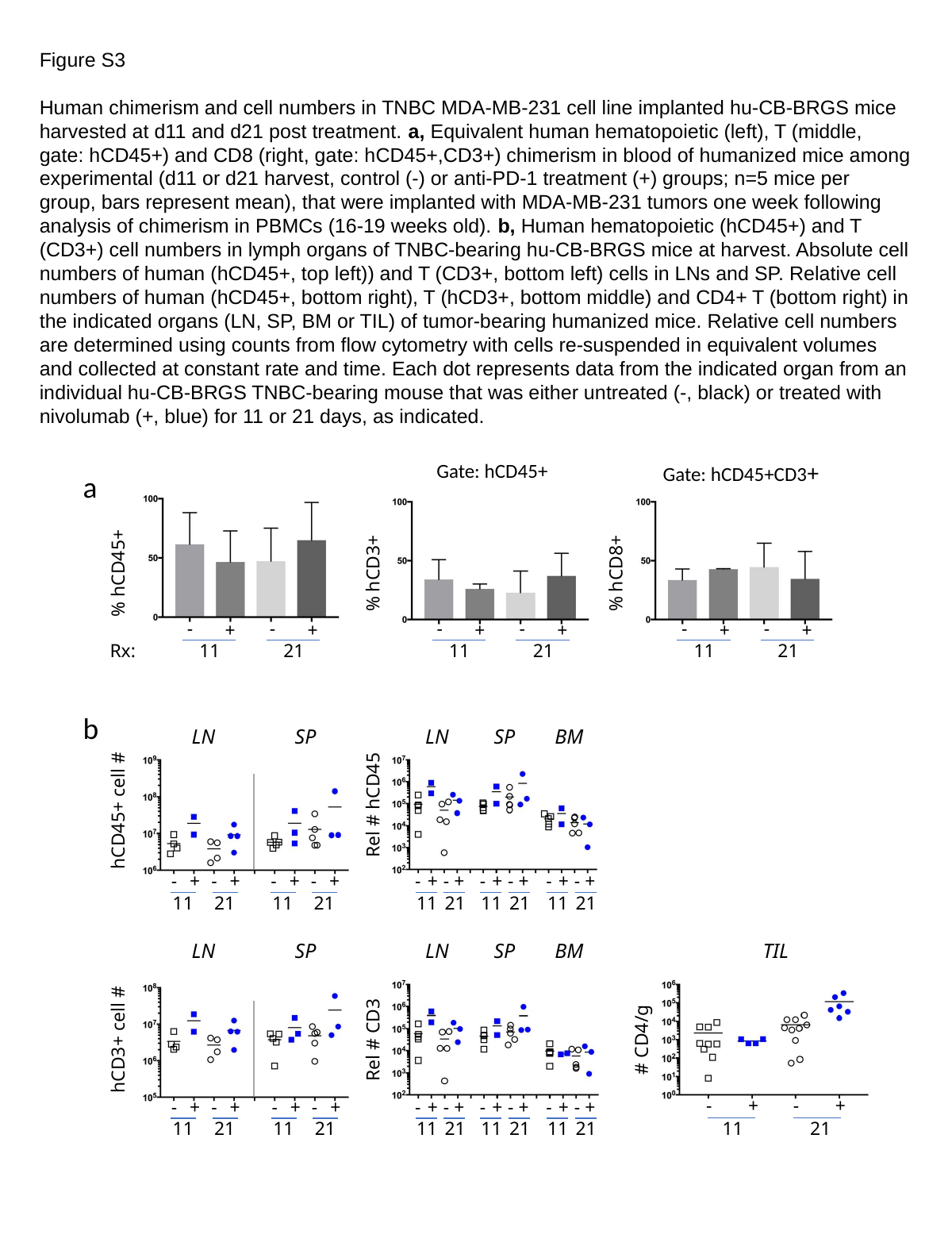

Figure S3
Human chimerism and cell numbers in TNBC MDA-MB-231 cell line implanted hu-CB-BRGS mice harvested at d11 and d21 post treatment. a, Equivalent human hematopoietic (left), T (middle, gate: hCD45+) and CD8 (right, gate: hCD45+,CD3+) chimerism in blood of humanized mice among experimental (d11 or d21 harvest, control (-) or anti-PD-1 treatment (+) groups; n=5 mice per group, bars represent mean), that were implanted with MDA-MB-231 tumors one week following analysis of chimerism in PBMCs (16-19 weeks old). b, Human hematopoietic (hCD45+) and T (CD3+) cell numbers in lymph organs of TNBC-bearing hu-CB-BRGS mice at harvest. Absolute cell numbers of human (hCD45+, top left)) and T (CD3+, bottom left) cells in LNs and SP. Relative cell numbers of human (hCD45+, bottom right), T (hCD3+, bottom middle) and CD4+ T (bottom right) in the indicated organs (LN, SP, BM or TIL) of tumor-bearing humanized mice. Relative cell numbers are determined using counts from flow cytometry with cells re-suspended in equivalent volumes and collected at constant rate and time. Each dot represents data from the indicated organ from an individual hu-CB-BRGS TNBC-bearing mouse that was either untreated (-, black) or treated with nivolumab (+, blue) for 11 or 21 days, as indicated.
Gate: hCD45+
Gate: hCD45+CD3+
a
% hCD45+
% hCD8+
% hCD3+
 -
 +
 -
 +
11
21
 -
 +
 -
 +
11
21
 -
 +
 -
 +
11
21
Rx:
b
LN
SP
LN
SP
BM
Rel # hCD45
hCD45+ cell #
-
+
-
+
11
21
-
+
-
+
 -
 +
 -
 +
 -
 +
 -
 +
 -
 +
 -
 +
11
21
11
21
11
21
11
21
LN
SP
LN
SP
BM
TIL
hCD3+ cell #
Rel # CD3
# CD4/g
-
+
-
+
-
+
-
+
11
21
-
+
-
+
11
21
 -
 +
 -
 +
 -
 +
 -
 +
 -
 +
 -
 +
11
21
11
21
11
21
11
21

## Slide 4
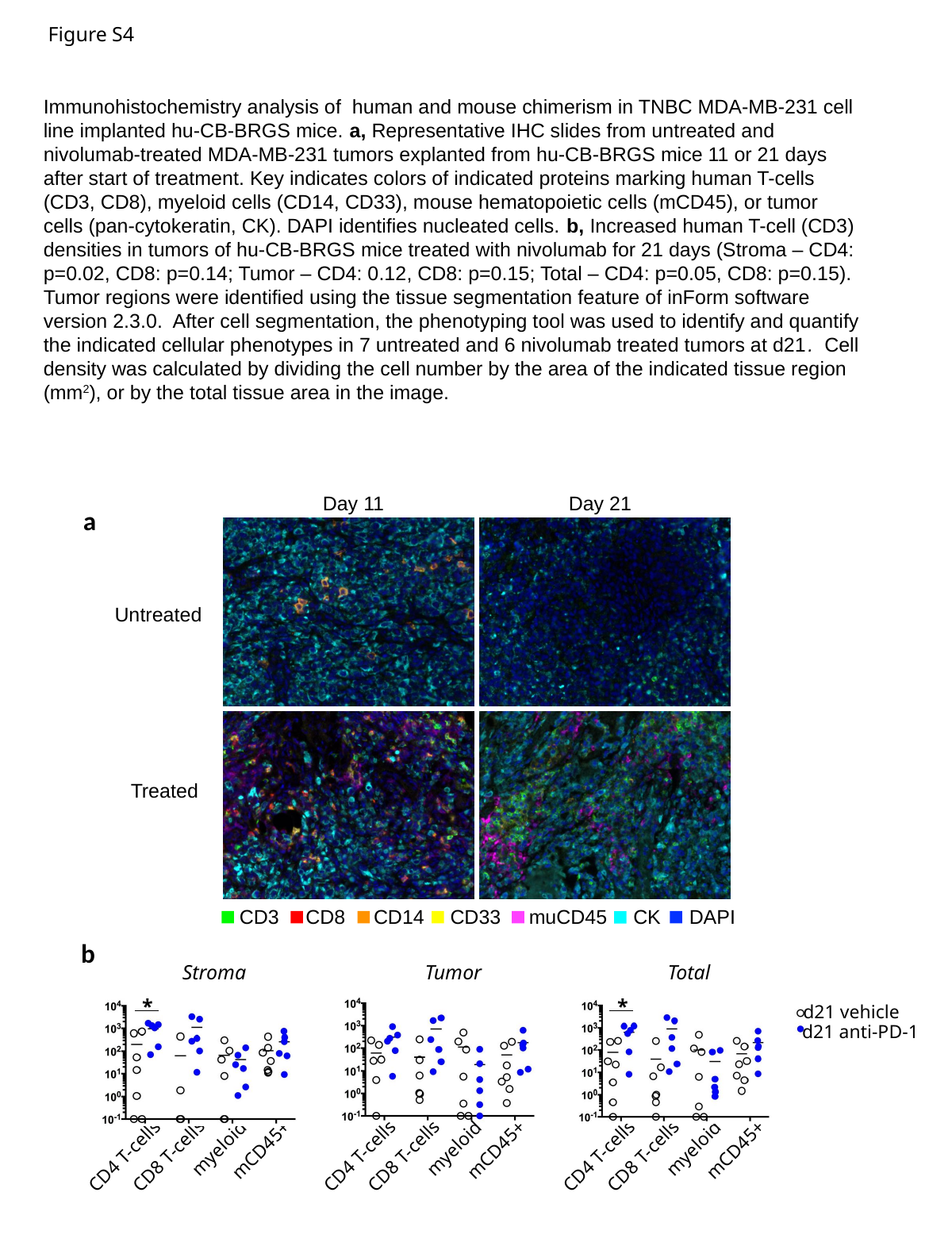

Figure S4
Immunohistochemistry analysis of human and mouse chimerism in TNBC MDA-MB-231 cell line implanted hu-CB-BRGS mice. a, Representative IHC slides from untreated and nivolumab-treated MDA-MB-231 tumors explanted from hu-CB-BRGS mice 11 or 21 days after start of treatment. Key indicates colors of indicated proteins marking human T-cells (CD3, CD8), myeloid cells (CD14, CD33), mouse hematopoietic cells (mCD45), or tumor cells (pan-cytokeratin, CK). DAPI identifies nucleated cells. b, Increased human T-cell (CD3) densities in tumors of hu-CB-BRGS mice treated with nivolumab for 21 days (Stroma – CD4: p=0.02, CD8: p=0.14; Tumor – CD4: 0.12, CD8: p=0.15; Total – CD4: p=0.05, CD8: p=0.15). Tumor regions were identified using the tissue segmentation feature of inForm software version 2.3.0. After cell segmentation, the phenotyping tool was used to identify and quantify the indicated cellular phenotypes in 7 untreated and 6 nivolumab treated tumors at d21. Cell density was calculated by dividing the cell number by the area of the indicated tissue region (mm2), or by the total tissue area in the image.
Day 11
Day 21
a
Untreated
Treated
CD3
CD8
CD14
CD33
muCD45
DAPI
CK
b
Stroma
Tumor
Total
myeloid
mCD45+
CD4 T-cells
CD8 T-cells
myeloid
mCD45+
CD4 T-cells
CD8 T-cells
myeloid
mCD45+
CD4 T-cells
CD8 T-cells
*
*
d21 vehicle
d21 anti-PD-1

## Slide 5
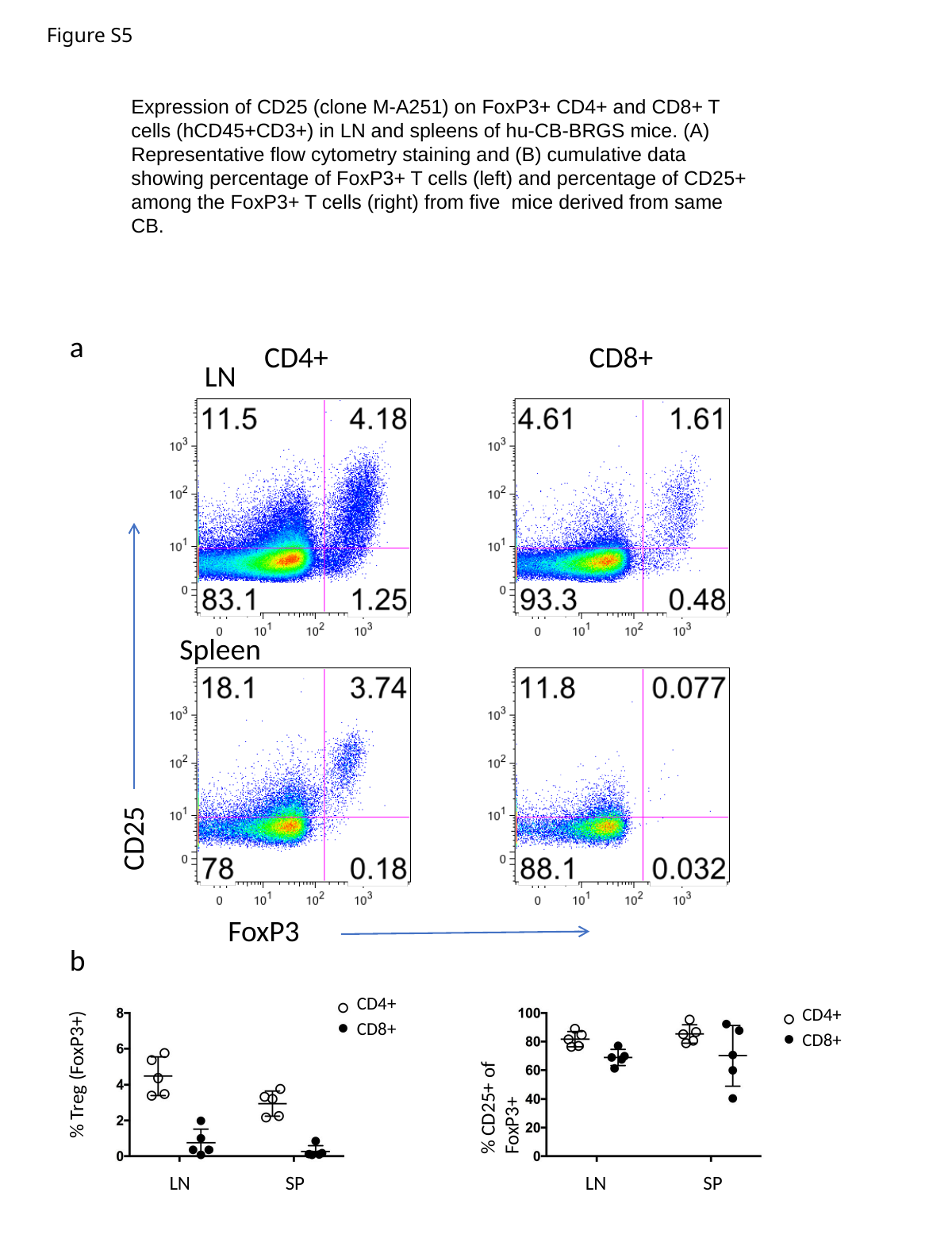

Figure S5
Expression of CD25 (clone M-A251) on FoxP3+ CD4+ and CD8+ T cells (hCD45+CD3+) in LN and spleens of hu-CB-BRGS mice. (A) Representative flow cytometry staining and (B) cumulative data showing percentage of FoxP3+ T cells (left) and percentage of CD25+ among the FoxP3+ T cells (right) from five mice derived from same CB.
a
CD4+
CD8+
LN
Spleen
CD25
FoxP3
b
% Treg (FoxP3+)
CD4+
% CD25+ of FoxP3+
CD4+
CD8+
CD8+
LN
SP
LN
SP

## Slide 6
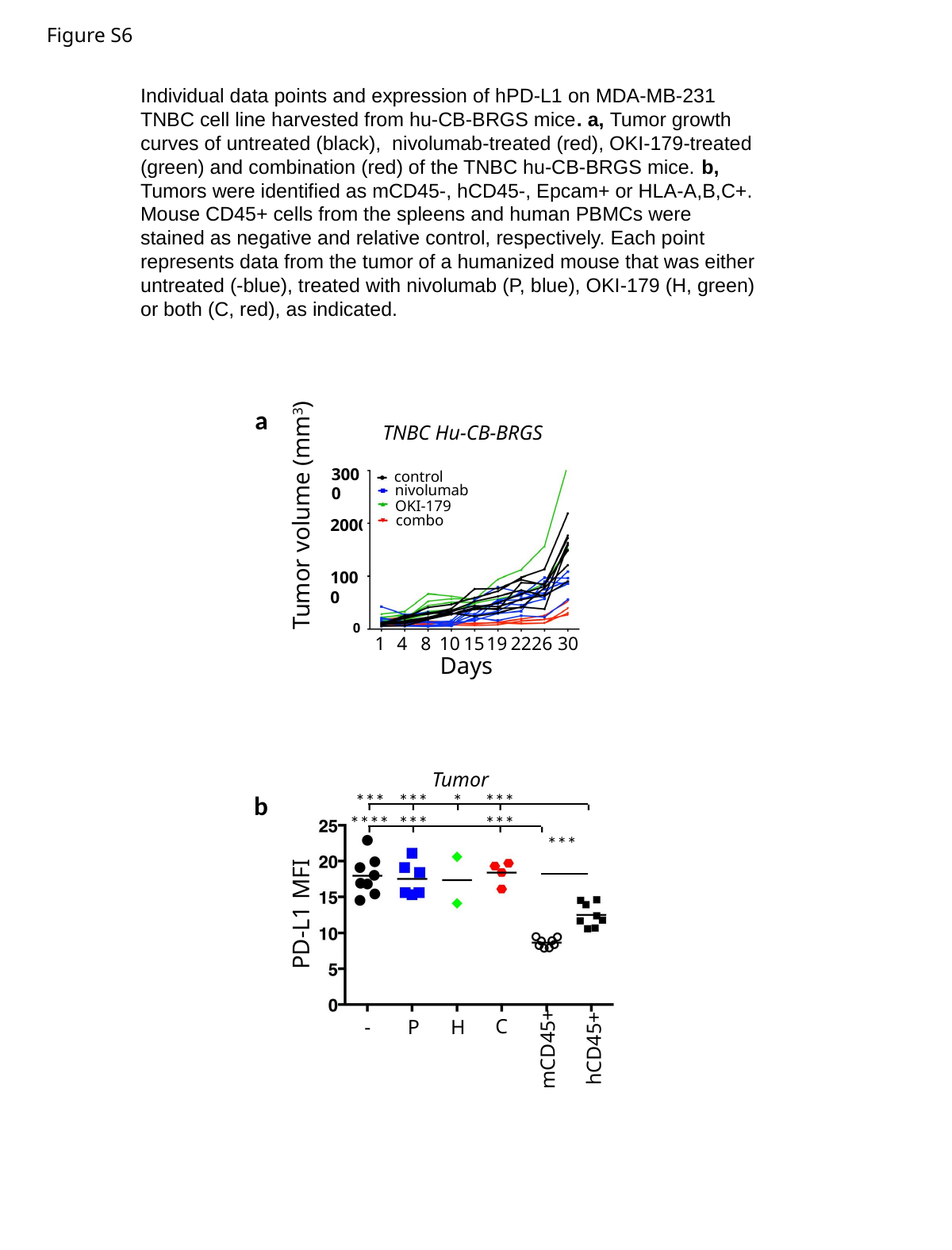

Figure S6
Individual data points and expression of hPD-L1 on MDA-MB-231 TNBC cell line harvested from hu-CB-BRGS mice. a, Tumor growth curves of untreated (black), nivolumab-treated (red), OKI-179-treated (green) and combination (red) of the TNBC hu-CB-BRGS mice. b, Tumors were identified as mCD45-, hCD45-, Epcam+ or HLA-A,B,C+. Mouse CD45+ cells from the spleens and human PBMCs were stained as negative and relative control, respectively. Each point represents data from the tumor of a humanized mouse that was either untreated (-blue), treated with nivolumab (P, blue), OKI-179 (H, green) or both (C, red), as indicated.
a
TNBC Hu-CB-BRGS
Tumor volume (mm3)
3000
control
nivolumab
OKI-179
combo
2000
1000
0
1
4
8
10
15
19
22
26
30
Days
Tumor
***
***
*
***
-
-
-
-
-
****
***
***
-
-
-
-
***
PD-L1 MFI
C
-
P
H
hCD45+
mCD45+
b

## Slide 7
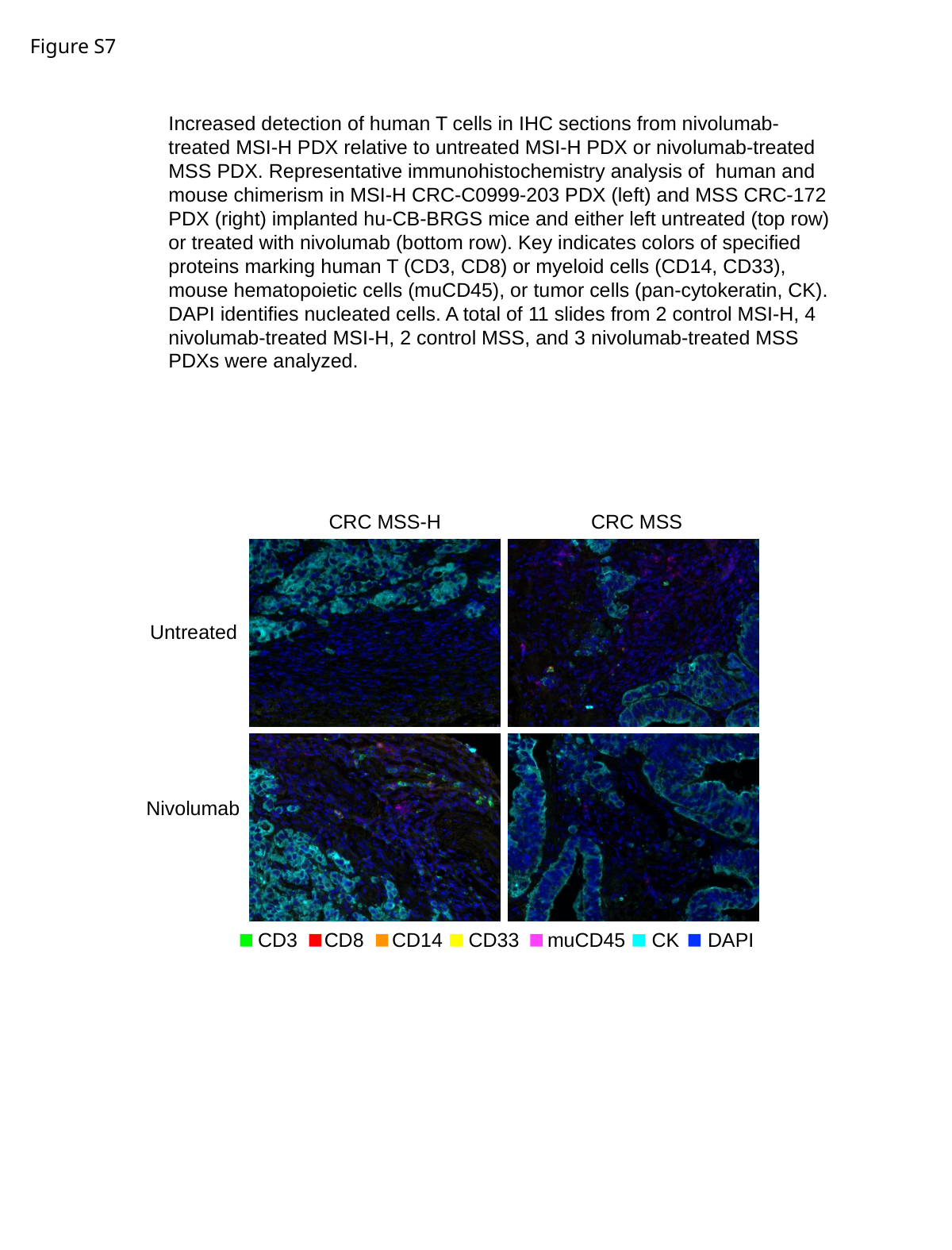

Figure S7
Increased detection of human T cells in IHC sections from nivolumab-treated MSI-H PDX relative to untreated MSI-H PDX or nivolumab-treated MSS PDX. Representative immunohistochemistry analysis of human and mouse chimerism in MSI-H CRC-C0999-203 PDX (left) and MSS CRC-172 PDX (right) implanted hu-CB-BRGS mice and either left untreated (top row) or treated with nivolumab (bottom row). Key indicates colors of specified proteins marking human T (CD3, CD8) or myeloid cells (CD14, CD33), mouse hematopoietic cells (muCD45), or tumor cells (pan-cytokeratin, CK). DAPI identifies nucleated cells. A total of 11 slides from 2 control MSI-H, 4 nivolumab-treated MSI-H, 2 control MSS, and 3 nivolumab-treated MSS PDXs were analyzed.
CRC MSS-H
CRC MSS
Untreated
Nivolumab
CD3
CD8
CD14
CD33
muCD45
DAPI
CK
